# Auricular Acupressure for Perioperative Anxiety and Pain in Therapeutic Breast Surgery: Protocol for a Randomized Clinical Trial

**DOI:** 10.2196/97469

**Published:** 2026-08-21

**Authors:** Mirela Bilc, Eva Beiner, Johanna Triana, Irene Rubio Rodríguez, Anja Birmanac-Pflüger, Holger Cramer, Marcela Winkler

**Affiliations:** 1 Institute of General Practice and Interprofessional Care, University Hospital Tübingen, Osianderstr. 5, Tübingen, 72076, Germany, 49 711 8101-3766; 2 Bosch Health Campus, Robert Bosch Center for Integrative Medicine and Health, Stuttgart, Germany; 3 Department of Integrative Medicine, Robert-Bosch-Hospital, Stuttgart, Germany; 4 Department of Gynecology and Obstetrics, Robert-Bosch-Hospital, Stuttgart, Germany

**Keywords:** breast cancer, breast surgery, anxiety, pain, National Acupuncture Detoxification Association, NADA protocol, acupressure, acupuncture, randomized clinical trial

## Abstract

**Background:**

Preoperative anxiety is common among women undergoing breast cancer surgery and is associated with impaired subjective well-being as well as adverse perioperative outcomes, including increased postoperative pain, nausea and vomiting, and higher analgesic requirements. While preliminary evidence suggests potential benefits of nonpharmacological interventions, including acupuncture, robust clinical evaluation of safe and feasible approaches in perioperative oncology is still needed. The National Acupuncture Detoxification Association (NADA) protocol, a standardized 5-point auricular approach, has been investigated for its potential stress-regulating effects in other clinical contexts but has not yet been systematically evaluated in perioperative breast cancer surgery. To the best of our knowledge, this is the first randomized controlled trial to evaluate whether NADA auricular acupressure, when added to standard preoperative care, reduces perioperative anxiety and pain in women with newly diagnosed breast cancer undergoing therapeutic surgery.

**Objective:**

The present study aims to evaluate the effects of NADA auricular acupressure on perioperative anxiety and pain in patients with a first diagnosis of breast cancer undergoing therapeutic breast surgery. In addition, we will exploratorily assess whether this intervention further impacts decisions regarding the initiation or use of other complementary and integrative medicine (CIM) therapies within the first 3 months following surgery.

**Methods:**

This prospective, 2-arm, parallel randomized controlled trial will enroll 66 women with newly diagnosed breast cancer undergoing therapeutic surgery. Participants will be randomized 1:1 to receive either NADA auricular acupressure in addition to standard preoperative care or standard preoperative care alone. The primary outcome is preoperative state anxiety measured prior to surgery (T1) using the State-Trait Anxiety Inventory-State. Secondary outcomes include postoperative anxiety, preoperative and postoperative pain, postoperative nausea and vomiting, analgesic and antiemetic medication use, and postoperative complications. The subsequent use of CIM at the 3-month follow-up will be an exploratory outcome. Analyses will follow the intention-to-treat principle using analysis of covariance, linear mixed-effects models, linear regression, and logistic regression as appropriate.

**Results:**

Recruitment began in March 2026. At the time of manuscript submission, 8 participants have been recruited. The target sample size (N=66) is expected to be reached by April 2027, with data collection completed by July 2027 and results anticipated in September 2027.

**Conclusions:**

Conducted under routine clinical conditions, this trial evaluates NADA auricular acupressure within a real-world perioperative oncology setting. The randomized design and standardized intervention protocol represent key methodological strengths, while the absence of blinding and a sham control constitute important limitations. By addressing a relevant gap in the current evidence base on NADA auricular acupressure in perioperative oncology, this study will inform clinical practice and guide future research.

## Introduction

Current estimates show that approximately 2.3 million women worldwide were diagnosed with breast cancer in 2022, making it the most frequently diagnosed cancer among women in most countries [1]. This substantial global burden is mirrored in Germany as well, where around 74,500 new cases are reported annually among women, with men accounting for only about 1% of all newly diagnosed breast cancer cases [2]. Alongside systemic therapies and radiotherapy, surgical intervention constitutes 1 of the 3 principal components of breast cancer treatment [3].

Psychological distress is highly prevalent in patients with breast cancer and represents a substantial component of the overall disease burden [4]. Beyond uncertainty regarding disease progression, the period immediately preceding surgery has been identified as a particularly vulnerable phase, commonly associated with heightened anxiety. Findings from a sample of newly diagnosed patients with breast cancer waiting for surgery indicate that nearly 40% showed clinically relevant symptoms of anxiety and around 25% reported significant depressive symptoms [5].

Preoperative anxiety in patients with breast cancer has been described as a multifaceted phenomenon characterized by recurring thoughts and negative emotions related not only to the cancer diagnosis itself but also to the anticipated surgical procedure and concerns about future treatment [6]. The associated involuntary changes in body image may further exacerbate psychological distress and anxiety [7,8]. This aspect may partly explain why anxiety levels have been shown to be significantly higher in women undergoing oncologic breast surgery compared to those undergoing cosmetic breast procedures, underscoring the additional psychological burden imposed by a cancer diagnosis [9]. Importantly, preoperative anxiety is not only a matter of psychological well-being but also of clinical relevance for perioperative outcomes. Elevated anxiety has been associated with higher rates of adverse postoperative events, including postoperative nausea and vomiting (PONV), as well as increased postoperative pain [10,11]. A recent meta-analysis further demonstrated significant associations between elevated preoperative anxiety and increased consumption of anesthetics and analgesics [12]. Taken together, these findings suggest that effective reduction of preoperative anxiety may contribute not only to enhanced subjective well-being during surgical treatment but also to improved postoperative outcomes.

Various pharmacological and nonpharmacological options are available to address preoperative anxiety, and choosing the most suitable approach requires careful consideration of the patient’s individual anxiety severity and overall clinical context [13]. Nonpharmacological interventions have increasingly been integrated into perioperative care, particularly due to their favorable safety profile and their potential to complement standard medical management. A recent meta-analysis evaluating nonpharmacological approaches, including aromatherapy, music therapy, and acupuncture, demonstrated a moderate effect size in reducing preoperative anxiety among women undergoing breast surgery [14]. With specific regard to acupuncture, further evidence supports its potential to reduce preoperative anxiety. A meta-analysis of 12 randomized controlled trials comprising 916 preoperative adult patients undergoing emergency or elective surgeries under general anesthesia demonstrated that acupuncture significantly decreased preoperative anxiety when compared to control groups receiving sham acupuncture, as measured by validated instruments such as the State-Trait Anxiety Inventory-State (STAI-S) and the Visual Analogue Scale [15]. Despite these encouraging findings, the overall quality of evidence was rated as low to moderate, highlighting the need for further well-designed, adequately powered randomized controlled trials. Among the included trials, only one study specifically enrolled patients undergoing breast cancer surgery, and this study was judged to have an unclear risk of bias. In addition, substantial heterogeneity in the acupuncture and acupressure protocols applied limits comparability across studies and complicates the interpretation of pooled results.

One potentially promising and standardized approach is the National Acupuncture Detoxification Association (NADA) protocol, a specific form of auricular acupuncture originally developed as an adjunctive treatment in addiction medicine. The protocol consists of stimulation of 5 defined auricular points (sympathetic, shen men, kidney, liver, and lung) and is intended to promote emotional stabilization and support autonomic regulation [16,17]. Since its introduction, its application has expanded to other health care contexts, including the management of sleep disturbances [18,19], pain [20], or stress management [21,22]. Of particular relevance to perioperative medicine is its presumed stress-regulating effect. It is hypothesized that the NADA protocol modulates the autonomic nervous system, particularly by reducing sympathetic overactivity and enhancing parasympathetic tone, thereby attenuating anxiety and stress-related physiological responses [23]. Despite encouraging findings in other clinical contexts, the NADA protocol has been insufficiently investigated in oncology, particularly in the perioperative setting. Preliminary studies and case reports suggest potential benefits for patients with cancer, including those with breast and prostate cancer, especially in the management of endocrine therapy-related side effects, sleep disturbances, fatigue, anxiety, and depressive symptoms, while showing a low risk of adverse events [24-26]. However, systematic investigations in oncological settings remain limited, and rigorously designed randomized controlled trials are required to more clearly establish its efficacy, particularly with regard to its stress-regulating effects.

The present study aims to evaluate the effects of NADA auricular acupressure on perioperative anxiety and pain in patients with a first diagnosis of breast cancer undergoing therapeutic breast surgery. In addition, we will exploratorily assess whether this intervention further impacts decisions regarding the initiation or use of other complementary and integrative medicine (CIM) therapies within the first 3 months following surgery.

## Methods

### Design

This study is a prospective, 2-arm, parallel randomized controlled trial designed to evaluate the effectiveness of auricular acupressure according to the NADA protocol (NADA) in addition to standard preoperative care compared with standard preoperative care alone (control [CTRL]) under routine clinical practice conditions. As the primary aim is to evaluate the effectiveness of adding auricular acupressure to usual care rather than to isolate its specific treatment effects and mechanisms of action, a sham acupressure control was not included. The study was prospectively registered on ClinicalTrials.gov (identifier NCT07423039) on January 20, 2026 (Multimedia Appendix 1). The pragmatic nature of the trial was further evaluated using the Pragmatic Explanatory Continuum Indicator Summary (PRECIS)-2 tool [27] (Checklist 1). The protocol will be reported following the SPIRIT (Standard Protocol Items: Recommendations for Interventional Trials) 2025 statement guideline for protocols of randomized trials [28] (Checklist 2).

### Setting and Recruitment

This study will be carried out at the Robert-Bosch-Hospital in Stuttgart, Germany. Participants will be recruited at the Department of Integrative Medicine at Robert-Bosch Hospital as part of routine clinical care. The study physician will inform potentially eligible patients about the opportunity to participate in the trial. Patients who express interest will receive sufficient information about the study. Comprehensive oral information about the study procedures, objectives, potential risks, and benefits will be provided by appropriately delegated personnel, who will answer all questions in a clear and understandable manner.

### Participants

Eligible participants are (1) female patients (2) aged 18 years or older with (3) newly diagnosed breast cancer who are scheduled to undergo therapeutic breast surgery at Robert-Bosch Hospital following initial diagnosis—written informed consent is required prior to study participation (4).

Patients will be excluded if (1) they have been diagnosed with an anxiety disorder within the previous 12 months or (2) if, in the opinion of the principal investigator, a psychological or mental condition precludes safe or appropriate participation in the study. Including participants with a diagnosed anxiety disorder may have introduced substantial variability in anxiety response due to underlying psychopathology and/or ongoing treatment. As such, this could act as a confounding factor, influencing preoperative anxiety beyond the transient, situational anxiety associated with surgery and limiting the internal validity of our study. Additional exclusion criteria include (3) the use of hearing aids; (4) the presence of skin irritation, inflammation, or eczema of the ear; (5) pregnancy; (6) insufficient proficiency in the German language; (7) unwillingness to permit storage and processing of personal health data as required by the study protocol; (8) missing or incomplete informed consent; and (9) prior acupuncture treatment (including NADA auricular acupuncture or acupressure) within the previous 3 months. According to the German S3 guideline on breast cancer [29] and the AGO Breast Committee recommendations for early breast [30], therapeutic surgery is typically scheduled within 3 to 6 weeks after histological confirmation of the diagnosis or 3 to 6 weeks after completion of neoadjuvant systemic therapy when indicated. Patients are usually admitted on the day before or the morning of surgery. Perioperative care is delivered in accordance with institutional protocols, including preoperative assessment by the anesthesia team, general anesthesia, multimodal analgesia, and risk-adapted PONV prophylaxis. The typical length of hospital stay is 1 to 3 days for breast-conserving surgery and 3 to 5 days for mastectomy, with or without reconstruction. All patients enrolled in the study receive this standard care regardless of group allocation.

### Sample Size Calculation

As this is, to the best of our knowledge, the first study investigating the effects of ear acupressure on preoperative anxiety, we opted for a clinically meaningful effect to be the most appropriate basis for the sample size calculation [31,32]. The sample size was determined to ensure adequate statistical power to detect a clinically important difference between groups on the (STAI-S) [33]. Based on an 8-point minimal clinically important difference [34] and assuming an SD of 10 based on previous data in similar populations [35], this amounts to a large effect size (Cohen *d*=0.8; *f*=0.4).

The sample size estimation was performed using G*Power (version 3.1, Heinrich Heine University Düsseldorf, Germany) [36] specifying a covariance analysis (ANCOVA) with the treatment group as the between-subject factor and the baseline STAI-S score as a covariate, a 2-sided significance level of 5%, and a statistical power of 80%. This yielded a required total sample size of 52 patients. To account for an anticipated dropout rate of 20%, a total of 66 participants will be enrolled in the study.

### Randomization and Blinding

Participants (N=66) will be enrolled and randomly assigned in a 1:1 ratio to either the intervention group (NADA) or the control group (CTRL). The random allocation sequence will be generated by an independent statistician who is not involved in participant recruitment, enrollment, or intervention delivery. The sequence will be created using the R (R Foundation for Statistical Computing) package “blockrand” [37] and implemented via the REDCap (Research Electronic Data Capture; Vanderbilt University) [38]. Restricted randomization with blocks of variable lengths will be used to ensure balanced group sizes throughout the recruitment period and reduce predictability of group assignment. Stratified randomization will be performed according to the type of breast surgery, with 2 predefined strata: breast-conserving therapy and mastectomy.

Allocation concealment will be ensured through centralized randomization within REDCap. The allocation sequence will be integrated in the secure electronic system and will not be accessible to investigators responsible for screening or enrolling participants.

Participants will be enrolled by the study physician. Randomization will be performed electronically in REDCap after completion of baseline data collection. The group assignment will be revealed only after a participant has been enrolled and a baseline assessment (T0) has been completed. Study personnel responsible for enrollment and allocation will not have access to the full randomization list or information regarding block sizes.

Due to the nature of the auricular acupressure intervention, blinding of participants and study personnel is not feasible. However, statistical analyses will be performed by statisticians who are blinded to group allocation.

### Intervention

#### Intervention Group: NADA Auricular Acupressure Plus Standard Care (NADA)

Participants allocated to the intervention group will receive standardized auricular acupressure according to the NADA protocol in addition to standard preoperative care.

The intervention (Checklist 3) will be administered using adhesive auricular acupressure patches containing a small gold-coated pellet designed to exert continuous pressure on predefined auricular acupuncture points. Gold-coated pellets will be used to minimize the risk of contact allergy. According to the NADA protocol, 5 specific points will be treated unilaterally following a standardized procedure: sympathetic, shen men, kidney, liver, and lung [17]. While the original NADA protocol was developed for group settings using bilateral needling, unilateral application with semipermanent pellets has been described for individual, nongroup settings [17]. In our study, unilateral application was chosen to keep one ear available for perioperative monitoring. The ear selected for treatment will be determined by patient preference. The laterality of the treated ear will be documented in the patient records as part of the intervention. Correct placement of the pellets will be verified by NADA-certified study personnel based on standard anatomical landmarks.

The intervention will be initiated prior to surgery. After application, participants will be instructed to keep the patches in place for 5 consecutive days. They will be asked to manually stimulate by pressing or massaging each pellet 3 times daily for 1 minute until a mild pressure sensation is perceived. Adherence to the intervention will be assessed during daily visits by study personnel through verification of pellet placement and documentation of any adverse events. In case of detachment, pellets will be replaced as needed. Participants discharged before the end of the 5-day application period will be instructed to remove the patches themselves on the fifth day, in accordance with the written instructions provided at the start of the intervention. In addition, according to the predefined intervention procedure, pellets will be replaced after 5 days if the patients are not discharged before. Intervention fidelity will be ensured through delivery of the intervention exclusively by NADA-certified personnel (physicians, medical assistants, or nursing staff) who have received formal training in the NADA protocol. In Germany, NADA certification requires completion of 2 sequential courses provided by NADA Deutsche Sektion e.V. [39], a basic course (basic I) and a supervision and final course (basic II), together with documented practical applications. The certificate is valid for 5 years, after which a refresher course is required for renewal. Physicians may perform acupuncture independently, whereas other health care professionals apply the NADA protocol under medical delegation, for which the certificate serves as the formal qualification. Auricular acupressure according to the NADA protocol is a noninvasive and low-risk intervention.

#### Control Group: Standard Care (CTRL)

Participants allocated to the control group will receive standard preoperative care according to institutional practice. No auricular acupressure will be administered prior to surgery. To promote fairness between study groups, participants in the control group will be offered NADA auricular acupressure after completion of the last study measurement on the day of hospital discharge.

No restrictions will be placed on standard hospital procedures in either study group during the study period.

### Intervention Discontinuation and Adherence

The intervention will be discontinued in the event of adverse reactions, participant requests, or if continuation is deemed inappropriate by the investigator for medical or safety reasons. Participants may withdraw from the study at any time without providing a reason and without consequences for their medical care.

To promote adherence, participants in the intervention group will receive verbal and written instructions detailing instructions regarding acupressure point stimulation and behavioral recommendations during the intervention period. In addition, participants will receive daily visits from study personnel during their hospital stay to reinforce instructions, address questions, and monitor proper application of the auricular patches and safety.

### Outcomes

**Figure 1.**
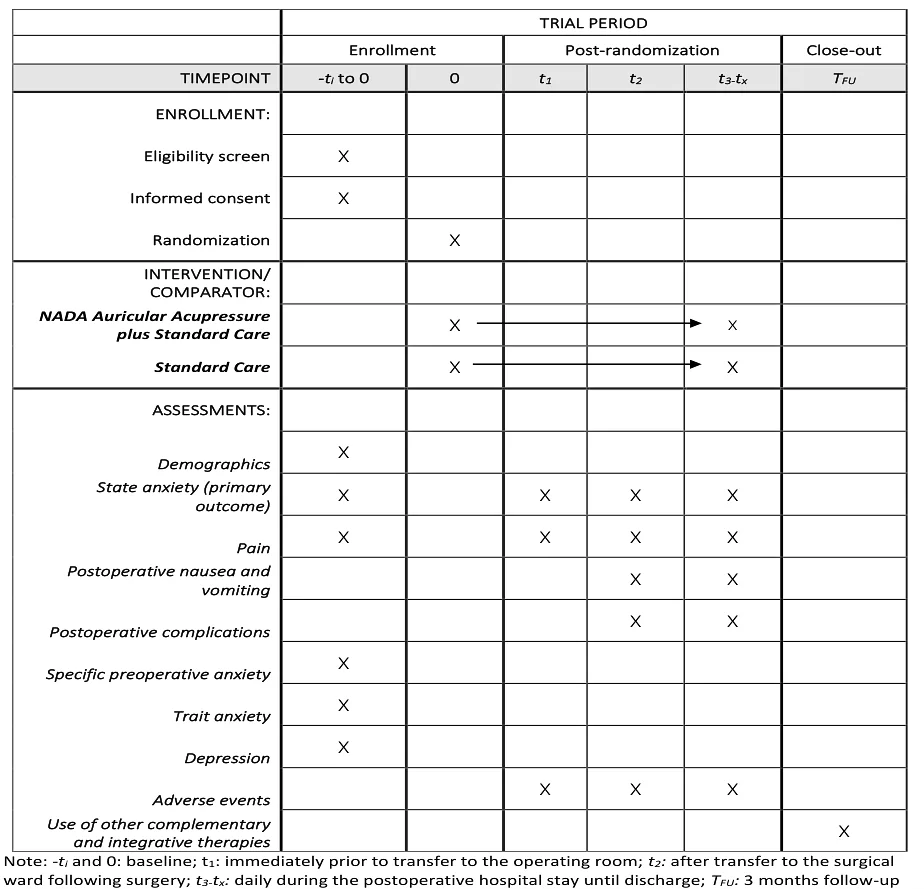
SPIRIT (Standard Protocol Items: Recommendations for Interventional Trials) 2025 participant timeline: schedule of enrollment, interventions, and assessments. NADA: The National Acupuncture Detoxification Association.

Data will be collected at the following time points: baseline (T0), prior to surgery (T1), after transfer to the surgical ward following surgery (T2), daily during the postoperative hospital stay until discharge or up to a maximum of 10 days, whichever occurs first (T3-Tx), and at 3-month follow-up (T_FU_). An overview schedule of enrollment, interventions, and assessments is presented in Figure 1 [28].

The primary outcome of this study is preoperative state anxiety prior to surgery (T1) measured using the State subscale of STAI-S [33].

Secondary outcomes include postoperative anxiety, preoperative and postoperative pain, PONV, use of analgesic and antiemetic medication, postoperative complications, specific surgery-related preoperative anxiety, trait anxiety, depressive symptoms, and therapy decisions at follow-up.

Postoperative anxiety will be measured using STAI-S after transfer to the surgical ward (T2) and daily during hospitalization (T3-Tx). Current pain intensity will be assessed using a Numerical Rating Scale (NRS; 0‐10) where higher scores represent higher pain intensity and will be measured both pre- (T0 and T1) and postoperative (T2-Tx). PONV will be assessed jointly using an NRS (0‐10) where higher scores represent higher symptom severity. Assessments will be performed after transfer to the surgical ward (T2) and daily during hospitalization (T3-Tx). This approach is consistent with current perioperative research and clinical practice, as nausea and vomiting are considered to share common risk factors and are addressed together in current clinical guidelines for the prevention and management of PONV [40,41]. For outcomes assessed daily during hospitalization, the same standardized questions were used at each assessment timepoint to ensure consistency across repeated measurements.

Analgesic consumption and the use of antiemetic medication will be extracted from medical records and summarized from surgery until discharge. Due to possible variability in prescribed agents and dose adjustments based on symptom severity, medication use will be summarized by drug class (eg, opioids, nonopioid analgesics, and antiemetics). Opioid consumption will be converted to oral morphine equivalents (OME) to allow standardized comparisons across participants [42]. Nonopioid analgesics and antiemetics will be summarized as cumulative dose within class and frequency of administration (eg, number of doses given).

The assessment window for postoperative complications extends from transfer to the surgical ward (T2) until hospital discharge. Accordingly, early postoperative complications were defined based on the current literature, including postoperative hematoma, seroma formation, surgical site infection, skin flap necrosis, nipple and nipple–areolar complex necrosis, venous thromboembolism, and nerve injuries [43]. Given the short hospital stay, only complications occurring during hospitalization will be captured; complications with a later typical onset, such as delayed wound infection or postmastectomy pain syndrome, are outside the assessment window of this trial. Data on postoperative complications will be extracted from patients’ medical records, documented, and summarized as incidence proportions.

Preoperative anxiety specifically related to surgery will be measured at baseline (T0) using the Amsterdam Preoperative Anxiety and Information Scale (APAIS) [44]. Trait anxiety will be assessed at baseline (T0) using the Trait subscale of the State-Trait Anxiety Inventory-Trait (STAI-T) [33]. Depressive symptom severity will be measured at baseline (T0) using the Beck Depression Inventory-II (BDI-II) [45]. Sociodemographic characteristics will be collected at baseline (T0) using a self-administered questionnaire. At the 3-month follow-up (T_FU_), participants’ decisions regarding the initiation or use of other CIM therapies will be assessed using a self-developed questionnaire.

### Data Management and Monitoring

Personal data will be collected and processed within this study in accordance with applicable data protection regulations. All study data will be documented and archived in pseudonymized form using a secure electronic database (REDCap). Access to the database is restricted to authorized study personnel, who are formally bound by professional confidentiality and data protection obligations. The pseudonymization key linking study IDs to personal identifiers will be stored separately in a password-protected electronic file accessible only to designated, nonblinded members of the study team.

No formal interim analyses are planned. Given the short intervention period and the low-risk profile of the intervention, no predefined stopping rules have been established. In addition to this, given the relatively small sample size, the establishment of an independent Data Monitoring Committee is not considered necessary. Study conduct, data quality, and protocol adherence will be monitored internally by the study team. Any protocol deviations or safety-relevant events will be documented and reviewed by the principal investigator.

### Safety

To evaluate safety, adverse events will be systematically assessed from initiation of the intervention until hospital discharge (T1-Tx) by actively inquiring about and documenting any undesirable effects. The severity of each adverse event will be classified as mild, moderate, severe, or life-threatening. In addition, a causality assessment will be performed for each event and categorized as no relationship, possible relationship, probable relationship, or definite relationship to the study intervention. The total number of adverse events and the number and percentage of participants experiencing at least 1 adverse event will be summarized by group.

### Data Analysis

Preoperative outcomes assessed at T1 (preoperative state anxiety and preoperative pain) will be analyzed using ANCOVA with the treatment group as the between-subject factor (NADA vs CTRL) and the respective baseline value as the covariate. Adjusted mean differences between groups and effect sizes (Cohen *d*) will be reported with corresponding 95% CIs. A 2-sided significance level of 5% will be applied.

In an exploratory analysis, the time interval between the T1 STAI-S assessment and surgery will be included as an additional covariate to account for potential variability in the timing of the anxiety assessment. Some variation in timing is expected due to the practical constraints of conducting the study in a clinical setting, and preoperative anxiety may increase as surgery approaches.

Repeated continuous postoperative outcomes T2-Tx (state anxiety, pain, PONV) will be analyzed using linear mixed-effects models including fixed effects for treatment group, time, and the group × time interaction, with participants included as a random effect.

Cumulative analgesic and antiemetic medication use from surgery to discharge will be compared between groups using linear regression models. If substantial violations of model assumptions are observed that cannot be addressed through appropriate data transformations, alternative analyses will be used, such as generalized linear models. Overall postoperative complication rates will be analyzed using logistic regression models and reported as odds ratios (ORs) with corresponding CIs.

Exploratory moderation analyses will be performed to evaluate whether baseline characteristics, including surgery-specific preoperative anxiety, trait anxiety, and depressive symptoms, modify the treatment effect on the primary outcome. Separate models will be fitted for each moderator as extensions of the primary ANCOVA model. Each model will include the baseline covariate, the treatment group, the respective moderator, and the group x moderator interaction term. A significant interaction term will be interpreted as evidence of a moderating effect.

All analyses will be conducted according to the intention-to-treat (ITT) principle. All randomized participants will be included in the analysis and analyzed according to their allocated group, irrespective of adherence to the intervention. Missing data will be handled using multiple imputation by chained equations (MICE) under the assumption that data are missing at random using the “mice” package for R [46]. Due to the nature of longitudinal data collection, between-participant variation in number of hospitalization days is expected and will be accounted for. Observations expected during hospitalization but not available will be imputed; however, measurements not collected because of hospital discharge will not be imputed. Analyses will be performed on multiply imputed datasets, and parameter estimates across the imputed datasets will be combined using Rubin’s rule.

As a sensitivity analysis, a per-protocol analysis will be conducted, including only participants who adhered to their allocated treatment. Protocol adherence will be defined a priori. Participants in the intervention group will be considered protocol-adherent if they received the ear acupressure prior to surgery and continued the intervention for the subsequent 4 days or until hospital discharge, whichever occurred first. Participants in the control group will be considered adherent if they did not receive ear acupressure before the end of primary data collection (Tx).

In addition, an exploratory exposure-based sensitivity analysis will be performed at 3 months follow-up to assess the association between exposure to ear acupressure and the use of other CIM therapies. To this end, participants will be classified according to actual exposure to ear acupressure, defined as receipt of ear acupressure either during the intervention phase or after discharge in the control group. ORs and corresponding 95% CIs will be calculated to compare the likelihood of using CIM therapies between participants exposed to ear acupressure and those not exposed.

No formal adjustment for multiplicity will be applied, as the trial has a single primary outcome for the confirmatory analysis. Findings for the secondary outcomes, as well as the moderation and sensitivity analyses, are considered subsidiary and exploratory [47].

### Ethical Considerations

This study adheres to the principles of the Helsinki Declaration. The study has been approved by the Ethics Committee at the State Medical Association of Baden-Württemberg (Landesärztekammer Baden-Württemberg) (F-2025‐104, 17.11.2025). Written informed consent will be obtained only after adequate explanation has been provided. Participation is voluntary, and patients may withdraw their consent at any time without providing reasons and without any consequences for their medical care. Following informed consent, eligibility will be determined by the study physician or appropriately delegated personnel according to the predefined inclusion and exclusion criteria.

## Results

Funding for the study was confirmed in April 2025. The study is planned for a total duration of 18 months. Based on approximately 200 breast surgeries performed annually at Robert-Bosch-Hospital and an anticipated recruitment rate of 30%, the target sample size of 66 participants is expected to be reached within approximately 12 months. Recruitment began on March 31, 2026. At the time of manuscript submission, 8 participants have been recruited. Recruitment is anticipated to conclude in April 2027, with data collection expected to be completed by July 2027. Study results are projected to be available in September 2027. There was no patient or public involvement in the development of the study design, selection of outcome measures, or planning of the research procedures.

## Discussion

The present trial aims to evaluate the effectiveness of auricular acupressure according to the NADA protocol in addition to standard preoperative care in reducing perioperative anxiety and pain in women with a first diagnosis of breast cancer undergoing therapeutic surgery. It is hypothesized that auricular acupressure will be superior to standard care in reducing perioperative anxiety and pain in this patient population. This hypothesis is in line with previous findings, including both emergency and elective surgeries in clinically heterogeneous populations [15], and initial evidence suggesting beneficial effects of the NADA protocol in oncological patients outside the surgical context [24-26].

This trial has several methodological strengths. To the best of our knowledge, this is the first randomized controlled trial to evaluate NADA auricular acupressure in the perioperative context of breast cancer surgery. By focusing on a standardized 5-point auricular protocol applied within a defined perioperative time frame, this study seeks to address the substantial variability in acupuncture techniques, point selection, and treatment duration from previous research. Furthermore, the use of this standardized protocol enhances reproducibility and may facilitate future implementation in routine clinical care if efficacy is demonstrated. The randomized controlled design with centralized allocation concealment reduces the risk of selection bias. Stratified randomization according to surgical procedure further minimizes potential confounding. Another strength of this trial is that it is conducted within a real-world perioperative oncology setting and embedded into routine clinical care. The intervention is delivered under pragmatic conditions, which enhances the external validity of the findings.

Several limitations must also be considered. Due to the nature of the intervention, blinding of participants and treating personnel is not feasible. This might introduce bias in the measurement of outcomes, particularly in the assessment of patient-reported outcomes such as anxiety and pain. To address this limitation, objective secondary outcomes, including medication use and postoperative complications, are assessed to provide clinically relevant endpoints less susceptible to reporting bias. Furthermore, the study does not include a sham control condition. The primary aim was to evaluate the efficacy of NADA auricular acupressure as an adjunct to routine care rather than to isolate specific acupoint-related effects. Consequently, the design does not allow differentiation between specific physiological effects of point stimulation and nonspecific effects related to expectation, therapeutic attention, or contextual influences. In addition, the sample size estimation was based on detecting a minimal clinically important difference in preoperative state anxiety as measured by the STAI-S. The study is therefore adequately powered for the primary endpoint but is not specifically powered to detect statistically significant differences in secondary outcomes. These should be considered exploratory, and effect estimates and corresponding CIs will be reported to facilitate interpretation of the magnitude and precision of observed differences.

Despite these limitations, the study addresses an important gap in the field of integrative oncology. There is growing interest among patients and health care providers in nonpharmacological strategies to improve perioperative well-being. However, integration of such approaches into clinical practice requires evidence from rigorously designed randomized trials. If NADA auricular acupressure proves effective in reducing perioperative anxiety and pain, it could represent a low-risk, low-cost, and easy-to-implement adjunct to standard care. Conversely, if no significant benefit is observed, the study will still provide important evidence to reconsider current assumptions regarding the role of auricular acupressure in perioperative oncology. Additionally, it will provide valuable information regarding effect sizes and variability in this patient population, thereby informing the planning of following studies.

## Supplementary material

10.2196/97469 Multimedia Appendix 1 Protocol version log.

10.2196/97469 Checklist 1 PRECIS checklist.

10.2196/97469 Checklist 2 SPIRIT checklist.

10.2196/97469 Checklist 3 TIDIeR checklist.

## Abbreviations

ANCOVA: analysis of covariance
APAIS: Amsterdam Preoperative Anxiety and Information Scale
BDI-II: Beck Depression Inventory-II
CIM: complementary and integrative medicine
CTRL: control group
ITT: intention-to-treat
MICE: multiple imputation by chained equations
NADA: National Acupuncture Detoxification Association
NRS: Numerical Rating Scale
OME: oral morphine equivalents
OR: odds ratio
PONV: postoperative nausea and vomiting
PRECIS: Pragmatic Explanatory Continuum Indicator Summary
REDCap: Research Electronic Data Capture
SPIRIT: Standard Protocol Items: Recommendations for Interventional Trials
STAI-S: State-Trait Anxiety Inventory-State
STAI-T: State-Trait Anxiety Inventory-Trait
TIDIeR: Template for Intervention Description and Replication
