# Supplementary material for: Auricular Acupressure for Perioperative Anxiety and Pain in Therapeutic Breast Surgery: Protocol for a Randomized Clinical Trial
Source: JMIR Res Protoc. 2026 Aug 21;15:e97469. doi: 10.2196/97469 (PMC13496320; doi:10.2196/97469)
Supplement: Supplementary file 1 [file resprot-v15-e97469-s001.pdf]

S 1

Protocol version

Version log	Summary of revisions made	Impact
Version 1.0, 10.09.2025	Initial version submitted to the Ethics Committee (EC).	The EC reviewed the study documents and issued a conditional approval, subject to implementation of the requested revisions. (14.10.2025)
Version 2.0, 24.10.2025	Added information on the randomization procedure to the study protocol.<br>Reduced the inclusion and exclusion criteria in the patient information sheet to essential information.<br>Added an MRI information sheet describing the composition of the acupressure pellets (MRI Pass).<br>Revised and corrected items of the Beck Depression Inventory and the sociodemographic questionnaire.<br>Implemented other minor wording changes as per EC request.	The EC reviewed the revised documents and approved the study without further conditions. (17.11.2025)
Version 3.0, 15.12.2025	Data collection method was changed from paper and pencil format to RedCap.	The EC acknowledged the submitted amendment. (12.01.2026)

German (original version)

Studienprotokoll für die berufliche Beratung

zur Vorlage bei der Ethik-Kommission bei der Landesärztekammer Baden-Württemberg

Variante A: Prospektive Untersuchung

Stuttgart,

Datum: 15.12.2025

Inhalt

Strukturierte Synopse der Studie 4
1. Projekttitel, Versionsnummer, Versionsdatum 6
2. Verantwortlichkeiten 6
3. Wissenschaftlicher Hintergrund 6
4. Projektziele 9
5. Endpunkte/Zielgrößen 9
6. Studienpopulation 10
7. Methodik und Durchführung 11
7.1 Studiendesign 11
7.2 Intervention 11
7.3 Erfassende Daten 12
7.4 Erfassung und Bewertung von unerwünschten Ereignissen 13
7.5 Dokumentation unerwünschter Ereignisse 13
7.6 Ablauf der Studie 13
8. Strahlenanwendung 14
9. Nutzen-Risiko-Abwägung 14
10. Biometrie 15
11. Datenmanagement und Datenschutz 16
12. Telemedizinische Verfahren 18
13. Bioproben 18
14. Unterschrift Studienleiter/in 18
15. Literaturverzeichnis 19

Strukturierte Synopse der Studie

Version & Datum	Version 3, 15.12.2025
Antragsteller	Dr. med. Marcela Winkler<br>Auerbachstraße 110, 70376 Stuttgart<br>+49 711 8101-2073<br>marcela.winkler@rbk.de
Studienleiter/in	Dr. med. Marcela Winkler<br>Auerbachstraße 110, 70376 Stuttgart<br>+49 711 8101-2073<br>marcela.winkler@rbk.de
Titel der Studie	Ohrakupressur zur Reduktion von perioperativer Angst und Schmerzen bei therapeutischen Brustoperationen nach der Erstdiagnose von Mamma-Karzinom
Akronym	**ROSA - R**eduktion von **O**perationsbezogener **S**chmerz und **A**ngst bei Mammakarzinom
Thema/Krankheitsbild	Die Krebsdiagnose, bevorstehende Behandlungen und mögliche körperliche Veränderungen durch Operationen stellen eine große Belastung dar. Rund 60–80 % der Patientinnen berichten vor einer Brust-OP über präoperative Ängste, die nicht nur das Wohlbefinden, sondern auch den Therapieverlauf negativ beeinflussen können.<br>Das NADA-Protokoll, eine standardisierte Form der Ohrakupunktur, wurde ursprünglich zur Suchtbehandlung entwickelt. Sein Nutzen bei Krebspatientinnen ist bislang wenig erforscht, jedoch deuten Erfahrungen aus anderen Bereichen auf vielversprechendes Potenzial in der Onkologie hin.
Zielsetzung	Ziel der Studie ist es, die Wirkung von Ohr-Akupressur auf perioperative Angst und Schmerzen bei Patientinnen mit erster Brustkrebsdiagnose, die eine therapeutische Brustoperation erhalten, zu untersuchen.<br>Unsere Hypothese ist, dass die Ohr-Akupressur nach dem NADA-Protokoll die Angst vor der Operation und die postoperativen Schmerzen reduziert.
Studienvorhaben	Nach der Feststellung der Einschlusskriterien werden die Teilnehmerinnen in zwei Gruppen randomisiert: Interventionsgruppe und Kontrolgruppe. Die Erhebung der Zielparameter erfolgt zu folgenden Zeitpunkten: Baseline (T0), unmittelbar vor Abruf in den OP-Raum (T1), postoperativ auf der Station (T2) sowie täglich bis zur Entlassung (T3–Tx). Drei Monate nach der Operation wird per Post ein Fragebogen zur langfristigen Therapiepräferenz verschickt (T-Follow-up).
Studienbedingte Maßnahmen	Die **Interventionsgruppe** erhält die Ohrakupressur nach dem NADA-Protokoll. Die Patientinnen bekommen vor der Operation Akupunkturpflaster an fünf festgelegten Punkten am Ohr. Diese Pflaster bleiben für fünf Tage auf der Haut. Die Patientinnen werden gebeten, die Kügelchen im Pflaster dreimal täglich zu drücken oder zu massieren.<br>Die **Kontrollgruppe** erhält die standardisierte präoperative Versorgung. Um die Motivation zur Teilnahme zu erhöhen, wird den Patientinnen aus der Kontrollgruppe die NADA-Akupressur nach der Entlassung angeboten.
Studiendesign	Prospektive randomisierte kontrollierte Studie<br>Aufgrund der Art der Ohrakupressur-Intervention können Patienten und Studienpersonal nicht verblindet werden. Statistiker werden verblindet.

Patientenkollektiv	<u>Einschlusskriterien:</u><br>• Alter > 18 Jahre<br>• Weibliches Geschlecht<br>• Mamma-Karzinom Patientinnen die nach Erstdiagnose am Robert-Bosch-Krankenhaus eine therapeutische Brust-OP erhalten<br>• schriftliche Einverständniserklärung<br><u>Ausschlusskriterien:</u><br>• In den letzten 12 Monate neu diagnostizierte Angststörung<br>• Eine psychische Verfassung die es nach Meinung des Prüfarztes nicht zulassen, dass der Patientin an der Studie teilnimmt<br>• Nutzung von Hörgeräten<br>• Hautirritationen oder Ekzeme im des Ohrs<br>• Schwangerschaft<br>• unzureichende Deutschkenntnisse<br>• Fehlende Bereitschaft zur Speicherung und Weitergabe der persönlichen Krankheitsdaten im Rahmen des Protokolls<br>• Fehlende oder inkomplette Einwilligungserklärung<br>• Erhalt einer Akupunkturbehandlung (einschließlich NADA-Ohrakupunktur oder Akupressur) in den letzten drei Monaten
Endpunkte	<u>Primärer Endpunkt:</u><br>• Angstniveau unmittelbar vor der Operation (STAI-s)<br><u>Sekundäre Endpunkte:</u><br>• Schmerz (NRS, Medikation)<br>• Postoperative Übelkeit und Erbrechen (Patientenakte)<br>• Postoperative Komplikationen (Patientenakte)<br>• Präoperativer Angst (APAIS)<br>• Eigenschaftangst (STAI-t)<br>• Unerwünschte Ereignisse<br>• Therapieauswahl
Fallzahlkalkulation Stichprobengröße	Ein MCID von 8 auf dem STAI entspricht d = 0,8 (f = 0,4). Für eine Kovarianzanalyse mit Interventionsgruppe als Zwischenfaktor und Baseline als Kovariante, einem zweiseitigen Signifikanzniveau von 5% und einer Power von 80% werden 52 Patientinnen benötigt. Unter Berücksichtigung einer Drop-out Rate von 20% werden n = 66 Probandinnen in die Studie eingeschlossen und 1:1 randomisiert werden.
Studiendauer	Die Studiendauer pro Patientin umfasst den gesamten stationären Aufenthalt (im Mittel 3 Tage) sowie eine anschließende dreimonatige Nachbeobachtung.<br>Die Gesamtstudienlaufzeit beträgt 18 Monate.
Anzahl der beteiligten Zentren	1 Studienzentrum<br>Robert Bosch Krankenhaus<br>Auerbachstr. 110<br>70376 Stuttgart

1. Projekttitel, Versionsnummer, Versionsdatum

Projekttitel:	Ohrakupressur zur Reduktion von perioperativer Angst und Schmerzen bei therapeutischen Brustoperationen nach der Erstdiagnose von Mamma-Karzinom
Akronym	**ROSA** – **R**eduktion von **O**perationsbezogener **S**chmerz und **A**ngst bei Mammakarzinom
Versionsnummer:	3
Versionsdatum:	15.12.2025

2. Verantwortlichkeiten

Antragsteller und Ansprechpartner für Ethikkommission	Dr. Marcela Winkler
Studienleiter/in	Dr. Marcela Winkler<br>Auerbachstraße 110, 70376 Stuttgart<br>+49 711 8101-2073<br>marcela.winkler@rbk.de
Co-Investigator	Dr. Mirela-Ioana Bilc<br>Auerbachstraße 112, 70376 Stuttgart<br>Telefon +49 711 8101-3766<br>mirela.bilc@bosch-health-campus.com
Studienärztin	Dr. Marcela Winkler
Weitere beteiligte klinische/wissenschaftliche Personen	Prof. Dr. Holger Cramer<br>Prof. Dr. Georg Sauer

3. Wissenschaftlicher Hintergrund

Mit einer Inzidenz von ca. 70.000 Neuerkrankungen pro Jahr ist Brustkrebs die in Deutschland häufigste Krebserkrankung der Frau (1). Nur 1% der Neudiagnosen betreffen Männer. Ein chirurgischer Eingriff ist hierbei neben medikamentöser und Strahlentherapie eine der drei wichtigsten Therapiesäulen.

Neben Unsicherheit über Krankheitsverlauf und Notwendigkeit einer operativen Therapie, stellt die Zeitperiode direkt vor Operation eine besonders häufig mit Angst verbundene Situation dar (2). Präoperative Angst wird von Patientinnen und Patienten in einer Inzidenz von 60-80% angegeben, wobei Größe, Länge und Invasivität der Operation nicht entscheidend für das Ausmaß der präoperativen Angst sind (3-5). Die situativ empfundene Angst ist hierbei deutlich stärker von der subjektiven Wahrnehmung der Bedrohung als dem objektiven Ausmaß der Gefährdung beeinflusst (6, 7). Bei Frauen führt eine Brustkrebsoperation oft zu Veränderungen des Körperbildes, was zusätzliche psychische Belastungen und Ängste verursacht (8, 9).

Angst als Basisemotion hat hierbei, über eine Aktivierung der Hypothalamus-Hypophysen-Nebennierenrinden-Achse (HHNR) sowie einer Steigerung des Sympathikotonus, auch direkt somatische Auswirkungen auf unser vegetatives sowie neuroendokrines System (10). Zusätzlich beeinflusst wird Angst über den sensorischen Input der Sinnesorgane, welcher über den Thalamus und thalamo-amygdaläre Bahnen die Amygdala erreicht. Diese ist entscheidend für Verarbeitung des sensorischen Inputs und über eine potentielle Weiterleitung an den Hypophysenvorderlappen auch für eine Aktivierung der HHNR-Achse. Die evolutionsbiologisch in Fluchtsituationen nötige Sympathikusaktivierung, welche durch Angst aktiviert wird, führt über die Katecholamine Noradrenalin und Adrenalin unter anderem zu einer Steigerung des Blutdrucks, der Herzfrequenz und des Herzminutenvolumens (10).

Diese Erhöhung führt vor allem bei kardiologisch vorbelasteten Patienten zu einer Erhöhung des Risikos für kardialen Ischämien, einem perioperativen Herzinfarkt oder Schlaganfall und bei Patienten mit vorbestehenden Aneurysmata über das Rupturrisiko zu einer erhöhten Mortalität (11, 12).

Für die Entfernung von Tumoren wird die Angstreaktion zusätzlich über eine Reduktion der Immunfunktion bei erhöhter Kortisolausschüttung relevant. Diese Kortisol bedingte Immunsupression führt somit neben einem erhöhten Risiko für postoperative Infektionen und Neoplasien auch zu einem erhöhten Risiko der Metastasierung durch geringere Eliminierung präoperativ verschleppter Mikrometastasen (13, 14).

Ein weiterer nachgewiesen durch präoperative Angst beeinflusster Parameter ist der von Patienten empfundene postoperative Schmerz, welcher neben empfundener Angst zu zusätzlichem Leid der operierten Patienten beiträgt (15). Eine aktuelle Metaanalyse hat

signifikante Zusammenhänge zwischen präoperativer Angst und erhöhtem Bedarf an Anästhetika und Analgetika gezeigt (16).

Eine Reduktion der Angst der Patienten kann somit über eine Verbesserung der hämodynamischen Stabilität, der Verbesserung der Immunfunktion und Wundheilung und einer Reduktion des postoperativen Schmerzes zu einem gesamt besseren postoperativen Outcome führen (6).

Es gibt viele verschiedene therapeutische Möglichkeiten, sowohl pharmazeutische als auch nicht-pharmazeutische, doch bei der Wahl der besten Vorgehensweise ist es entscheidend, das Angstniveau des Patienten und die gesamte klinische Situation zu berücksichtigen (17). Akupunktur scheint eine vielversprechende, mit geringem Risiko verbundene Option zur Reduzierung von präoperativer Angst zu sein. Eine aktuelle Metaanalyse nicht-pharmakologischer Therapien, darunter Aromatherapie, Musik und Akupunktur, hat eine moderate Effektgröße bei der Reduzierung präoperativer Angst bei Patientinnen vor Brustoperationen festgestellt (18). Eine Metaanalyse (19) von 12 randomisierten kontrollierten Studien mit insgesamt 916 Patienten hat gezeigt, dass Akupunktur die präoperative Angst, gemessen mit der State-Trait Anxiety Inventory Scale (STAI-S) und der visuellen Analogskala (VAS), signifikant reduzieren kann. Die Qualität der Evidenz war jedoch niedrig bis moderat, was darauf hindeutet, dass weitere hochwertige Studien erforderlich sind, um diese Ergebnisse zu bestätigen. Außerdem wurden in diesen Studien verschiedene Akupunktur- oder Akupressurpunkte verwendet, was die Vergleiche erschwert.

Eine Methode, die hierbei vielversprechend sein könnte, ist das NADA-Protokoll, eine standardisierte Form der Ohrakupunktur, die ursprünglich als Begleittherapie in der Suchtmedizin entwickelt wurde. Es konnte jedoch in verschiedenen Kontexten, darunter Katastrophenmedizin (z. B. nach den Terroranschlägen von 9/11 oder dem Hurrikan Katrina) sowie in der Betreuung traumatisierter Menschen, seine Wirksamkeit unter Beweis stellen. Besonders relevant für die perioperative Medizin ist die stressregulierende Wirkung des NADA-Protokolls. Es wird vermutet, dass durch eine Modulation des autonomen Nervensystems, insbesondere über eine Reduktion der sympathischen Überaktivität und eine Stärkung des parasympathischen Systems, Angst und stressbedingte physiologische Reaktionen abgemildert werden können (20).

Das NADA-Protokoll ist speziell im Kontext Onkologie aber bislang nur unzureichend untersucht, obwohl die Erfahrungen aus anderen Therapieindikationen einen potentiellen Nutzen für onkologische Patienten vermuten lassen. Erste Studien und Fallberichte zeigen positive Effekte bei onkologischen Patienten, insbesondere bei Brustkrebs- und Prostatakrebspatienten, die unter Nebenwirkungen endokriner Therapien, Schlafstörungen, Fatigue, Angst und depressiver Symptomatik leiden.

Allerdings gibt es bisher kaum systematische Untersuchungen zur Anwendung des NADA-Protokolls im onkologischen Kontext, insbesondere im perioperativen Setting. Die bisherigen Studien weisen auf mögliche positive Effekte hin, jedoch sind gute randomisierte kontrollierte Studien erforderlich, um die genaue Wirksamkeit und den optimalen Einsatzbereich in diesem Setting zu evaluieren.

4. Projektziele

Diese Studie untersucht die Wirkung von Ohr-Akupressur auf perioperative Angst und Schmerzen bei Patientinnen nach erster Brustkrebsdiagnose, die eine therapeutische Brustoperation erhalten. Konkret werden wir die Wirkung des NADA-Akupressurprotokolls auf das präoperative Angstniveau und postoperative Schmerzen untersuchen. Wir werden auch explorativ untersuchen, ob diese Intervention die Therapieauswahl in den folgenden 3 Monaten nach der Operation beeinflusst.

5. Endpunkte/Zielgrößen

Der primäre Zielparameter dieser Studie ist das Angstniveau unmittelbar vor der Operation, das anhand des State-Trait Anxiety Inventory (STAI-s) gemessen wird. Sekundäre Zielparameter umfassen die Schmerzintensität, die Postoperative Übelkeit und Erbrechen (PONV), postoperative Komplikationen, die spezifische präoperative Angst sowie unerwünschte Ereignisse. Die Therapieauswahl wird nach 3 Monaten Follow-up anhand eines per Post versandten Fragebogens erhoben.

Unsere Hypothese ist, dass die Ohr-Akupressur nach dem NADA-Protokoll die Angst vor der Operation und die postoperativen Schmerzen reduziert.

6. Studienpopulation

Für diese Studie werden wir N = 66 Mamma-Karzinom Patientinnen rekrutieren, die nach Erstdiagnose am Robert-Bosch-Krankenhaus eine therapeutische Brust-OP erhalten. Die Rekrutierung erfolgt direkt über die Abteilung für Naturheilkunde und Integrative Medizin im am Robert-Bosch-Krankenhaus.

Im Rahmen der routinemäßigen Versorgung informiert die/der Studienärzt*in* potenzielle Teilnehmerinnen über die Möglichkeit einer Studienteilnahme. Bei bestehendem Interesse erhalten Patientinnen die Patienteninformation zur Studie. Die/der Studienärzt*in* klärt die Patientinnen umfassend über die Studie auf und beantwortet alle auftretenden Fragen ausführlich und verständlich. Erst nach dieser Aufklärung wird die schriftliche Einwilligungserklärung zur Studienteilnahme eingeholt. Die Teilnahme erfolgt freiwillig und kann jederzeit ohne Angabe von Gründen widerrufen werden, ohne dass den Patientinnen daraus Nachteile entstehen.

Im Anschluss an die Einwilligung erfolgt ein medizinisches Screening durch den/die Studienärztin, um zu prüfen, ob die jeweilige Patientin die Voraussetzungen für eine Studienteilnahme erfüllt. Die Entscheidung über die Einschlussfähigkeit erfolgt anhand der folgenden Kriterien:

Einschlusskriterien:

- Alter > 18 Jahre
- Weibliches Geschlecht
- Mamma-Karzinom Patientinnen die nach Erstdiagnose am Robert-Bosch-Krankenhaus eine therapeutische Brust-OP erhalten
- schriftliche Einverständniserklärung

Ausschlusskriterien:

- In den letzten 12 Monate neu diagnostizierte Angststörung
- Eine psychische Verfassung die es nach Meinung des Prüfarztes nicht zulassen, dass der Patientin an der Studie teilnimmt
- Nutzung von Hörgeräten

- Hautirritationen oder Ekzeme des Ohrs
- Schwangerschaft
- unzureichende Deutschkenntnisse
- Fehlende Bereitschaft zur Speicherung und Weitergabe der persönlichen Krankheitsdaten im Rahmen des Protokolls
- Fehlende oder inkomplette Einwilligungserklärung
- Erhalt einer Akupunkturbehandlung (einschließlich NADA-Ohrakupunktur oder Akupressur) in den letzten drei Monaten

7. Methodik und Durchführung

7.1 Studiendesign

Bei dieser Studie handelt es sich um eine prospektive randomisierte kontrollierte Studie, bei der die Wirkung einer Ohrakupressur nach dem NADA-Protokoll mit einer Kontrollgruppe verglichen wird, die eine standardmäßige präoperative Versorgung erhält. Aufgrund der Art der Ohrakupressur-Intervention können Patienten und Studienpersonal nicht verblindet werden. Statistiker werden verblindet.

7.2 Intervention

Interventionsgruppe: NADA Ohrakupressur + Standardversorgung

Die Studienteilnehmenden im Interventionsarm erhalten zusätzlich zur Standardversorgung eine standardisierte Ohr-Akupressurbehandlung nach dem NADA-Protokoll. Diese wird mit speziellen Akupunkturpflastern durchgeführt, die ein kleines Kügelchen auf definierte Punkte am Ohr drücken. Im Rahmen des NADA-Protokolls werden fünf festgelegte Punkte an beiden Ohren nach einem standardisierten Konzept behandelt: Shen Men, Niere, Lunge, Leber und Vegetativum (24). Um eine Kontaktallergie zu vermeiden, werden Gold-beschichtete Kügelchen zur Anwendung kommen

Die Patienten erhalten das NADA-Protokoll vor der Operation. Sie werden gebeten, die Pflaster nach dem Anbringen für jeweils 5 Tage an den Ohren zu belassen und die Kügelchen eigenständig gemäß der mitgegebenen Anleitung dreimal täglich zu drücken oder zu massieren. Zu Beginn der Intervention erhalten die Patientinnen ein Informationsblatt mit Anwendungshinweisen und Verhaltensrichtlinien. (siehe Anlage 1).

NADA Ohrakupressur ist eine nicht-invasive und risikoarme Intervention. Zudem wird das Protokoll ausschließlich von NADA zertifizierten Ärzten, medizinische Fachangestellten oder Pflegepersonal durchgeführt

Kontrollgruppe: Standardversorgung

Patientinnen aus der Kontrollgruppe erhalten die präoperative Standardversorgung. Am Tag der Entlassung aus dem Krankenhaus wird ihnen die NADA-Akupressur angeboten.

7.3 Erfassende Daten

Allgemeine soziodemografische Daten werden zum Studienbeginn mittels Fragebogen erhoben. Diese Fragen umfassen unter anderem Familienstand, Herkunft, berufliche Situation und Krankheitstage.

<u>Primärer Zielparameter:</u>

Der primäre Zielparameter dieser Studie ist das Angstniveau unmittelbar vor der Operation, das anhand des State-Trait Anxiety Inventory (STAI-s) gemessen wird (21).

<u>Sekundäre Zielparameter</u>

Schmerz: Die Schmerzintensität wird anhand einer Numerischen Rating-Skala sowie der Medikation des Patienten aus der Patientenakte erfasst.

Postoperative Übelkeit und Erbrechen (PONV) sowie die Einnahme von Antiemetika werden aus der Patientenakte extrahiert. Zudem werden postoperative Komplikationen (z.B. Infekte, Nachblutungen) erfasst.

Präoperative Angst wird spezifisch anhand der Amsterdam Preoperative Anxiety and Information Scale (APAIS) erfasst (22).

Eigenschaftsangst wird mit dem State-Trait Anxiety Inventory (Trait-Messung) erfasst (21).

Die Depressionsausprägung wird präoperativ anhand des Beck-Depressionsinventar (BDI-II) erfasst (23).

Unerwünschte Ereignisse: Um die Sicherheit der Interventionen zu erfassen, werden unerwünschte Ereignisse während der ganzen Intervention systematisch erhoben, indem sie gezielt abgefragt und protokolliert werden.

Die Therapieauswahl wird nach 3 Monaten Follow-up anhand eines per Post versandten Fragebogens erhoben.

Um die Sicherheit der Interventionen zu erfassen, werden unerwünschte Ereignisse nach Intervention systematisch erfasst indem sie gezielt abgefragt und protokolliert werden.

7.4 Erfassung und Bewertung von unerwünschten Ereignissen

Unerwünschte Ereignisse sind Erkrankungen, Krankheitszeichen oder Symptome, die nach Einschluss des Patienten in die Studie während oder kurzzeitig nach Intervention eintreten oder sich verschlechtern. Die Ausprägung wird wie folgt bewertet: gering, mäßig, schwer, lebensbedrohend.

Für jedes Ereignis ist eine Kausalitätsbewertung vorzunehmen: kein Zusammenhang, möglicher Zusammenhang, wahrscheinlicher Zusammenhang, sicherer Zusammenhang.

Gemäß der GCP-Verordnung (GCP-V §3) ist „schwerwiegend“ wie folgt definiert: Schwerwiegendes unerwünschtes Ereignis oder schwerwiegende Nebenwirkung ist jedes, dass oder die: tödlich oder lebensbedrohend ist, eine stationäre Behandlung oder deren Verlängerung erforderlich macht, zu bleibender oder schwerwiegender Behinderung oder Invalidität führt oder eine kongenitale Anomalie oder einen Geburtsfehler zur Folge hat

7.5 Dokumentation unerwünschter Ereignisse

Jedes unerwünschte Ereignis ist zu dokumentieren, wenn nach Meinung des Prüfers ein ursächlicher Zusammenhang mit der Intervention besteht. Die Dokumentation umfasst die Art des Ereignisses, Schweregrad, Beginn, Dauer, Kausalität und Veränderungen für die Studie.

Im Zusammenhang stehende Krankheitszeichen und Symptome sollen zu einer einzigen Erkrankung zusammengefasst werden. Für die Dokumentation steht der Bogen „Unerwünschte Ereignisse Studienpatient“ zur Verfügung. Alle unerwünschten Ereignisse sind bis zum Abklingen oder bis zur Stabilisierung zu verfolgen.

7.6 Ablauf der Studie

Die Daten werden zu folgenden Zeitpunkten erhoben: Baseline (T0), unmittelbar vor Abruf im OP-Raum (T1), auf der Station nach OP (T2), täglich bis zur Entlassung (T3-Tx). Nach 3 Monaten wird ein Fragebogen zur Therapieauswahl per Post verschickt ($T_{Follow\text{-}up}$). Die Datenerhebung ist in Tabelle 1 dargestellt.

Tabelle 1: Ablauf der Datenerhebung

	T0	T1	T2	T3-Tx	$T_{Follow-up}$
STAI-s	x	x	x	x	
NRS Schmerz	x	x	x	x	
PONV			x	x	
Postoperative Komplikationen			x	x	
APAIS	x				
STAI-t	x				
BDI	x				
AE		x	x	x	
Fragebogen zur Therapieauswahl					x

Die Studie ist auf eine Laufzeit von 18 Monaten angelegt. Unter Berücksichtigung der Anzahl der Patientinnen, die jährlich am RBK eine Brustoperation erhalten (ca. 200) und einer Rekrutierungsquote von etwa 30% werden wir voraussichtlich in etwa 12 Monaten unsere erwartete Anzahl von N = 66 erreichen.

8. Strahlenanwendung

In Rahmen dieser klinischen Prüfung ist keine Strahlenanwendung vorgesehen. Es werden weder diagnostische noch therapeutische Verfahren eingesetzt, die mit Strahlenexposition für die Teilnehmenden verbunden sind.

9. Nutzen-Risiko-Abwägung

Die Teilnahme an der vorliegenden Studie kann für einzelnen Probandinnen mit potenziellen individuellen Nutzen verbunden sein, insbesondere durch eine mögliche Reduktion präoperativer Angst und Schmerzen. Darüber hinaus ist eine Gruppen- bzw. Fremdnutzen in Form eines wissenschaftlichen Erkenntnisgewinns zu erwarten, welcher zur Verbesserung zukünftiger präoperativer Behandlungsstrategien beitragen kann.

Die mit der Studienteilnahme verbundenen Belastungen und Risiken sind als gering einzuschätzen. Die Ohrakupressur ist eine nicht-invasive Intervention mit einem äußerst geringen Risikoprofil. Gelegentlich kann es zur Hautreizung kommen, ansonsten sind jedoch

keine negativen Nebenwirkungen beschrieben. Befragungen mittels Fragebogen bzw. Bewertungsskala sind auch vollkommen nicht-invasiv und weisen keinerlei studien-spezifische Risiken auf, der Zeitaufwand beträgt pro Befragung nur wenige Minuten.

Aus ärztlich-ethischer Sicht ist die Durchführung der Studie unter Berücksichtigung des derzeitigen Kenntnisstandes vertretbar, da der potenzielle Nutzen die zu erwartenden minimalen Risiken überwiegt. Die Teilnahme einzelner Probanden kann beendet werden bei unerwarteten Nebenwirkungen, Rückzug der Einwilligung oder aus medizinischen Gründen. Die vorzeitige Beendigung der gesamten Studie erfolgt bei unvorhergesehenen Sicherheitsbedenken, schwerwiegenden Komplikationen oder unzureichende Rekrutierung.

10. Biometrie

Gruppenunterschiede von mindestens 8 Punkten auf der STAI können als minimal klinisch bedeutsam angesehen werden (24). Legt man eine Standardabweichung von SD = 10 entsprechend dieser Population (25) zugrunde ergibt sich eine Effektstärke von d = 0.8. Ausgehend von einer entsprechenden Effektstärke von f = 0.4 ergibt eine Kovarianzanalyse mit Interventionsgruppe als Zwischenfaktor und Baseline als Kovariante, einem zweiseitigen Signifikanzniveau von 5% und einer Power von 80% eine benötigte Stichprobengröße von insgesamt 52 Patienten. Unter Berücksichtigung einer Drop-out Rate von 20% werden n = 66 Probandinnen in die Studie eingeschlossen und im Verhältnis 1:1 den Studiengruppen zugeordnet. Die Gruppenzuteilung erfolgt durch Blockrandomisierung mit variierenden Blocklängen sowie der Art der Brust-OP als Stratifikationsvariable. Die Randomisierung wird mittels einer computer-generierten Randomisierungsliste über das Datenerfassungstool REDCap (Research Electronic Data Capture) durchgeführt (26). Der Zeitpunkt der Randomisierung erfolgt nach der Baseline Erhebung T0.Die Auswertung des primären Zielparameters erfolgt auf der Basis des Intention-to-treat-Prinzips. Fehlende Werte werden multipel imputiert. Der primäre Zielparameter wird mit Hilfe einer univariaten Kovarianzanalyse (ANCOVA) konfirmatorisch analysiert, mit der Interventionsgruppe (NADA Ohrakupressur vs. Kontrolle) als Between-Faktor und der Baseline-Messung als Kovariate. Die sekundären Endpunkte werden explorativ mit denselben Modellen bewertet. Die Auswirkungen von Eigenschaftsangst und situativer präoperativer Angst werden durch Moderationsanalyse untersucht.

Als Maß für die Sicherheit und Akzeptanz der Intervention wird die Anzahl der Patientinnen, bei denen schwerwiegende unerwünschte Ereignisse oder nicht schwerwiegende unerwünschte Ereignisse auftreten oder die während des Studienzeitraums zur Nachbeobachtung ausscheiden, erhoben und mittels absoluter und relativer Häufigkeiten zwischen den Gruppen verglichen.

11. Datenmanagement und Datenschutz

Im Rahmen der Studie werden personenbezogenen Daten erhoben und verarbeitet. Die Dokumentation der Daten und deren Archivierung erfolgt pseudonymisiert in einer geschützten elektronischen Datenbank (REDCap), zu der nur befugte Mitarbeiterinnen und Mitarbeiter einschließlich auf das Berufs- und Datengeheimnis verpflichtete Doktorandinnen und Doktoranden Zutritt haben.

Als Datenplattform wird das Datenerfassungstool *REDCap* (Research Electronic Data Cap-ture) genutzt. *REDCap* ist eine kostenlose, sichere und browserbasierte Software zur Erstellung und Verwaltung von Datenbanken, die an der Vanderbilt University entwickelt und unter dem sog. *REDCap Consortium* verwaltet und vertrieben wird (Harris et al., 2009). *REDCap* kann auf mobilen Endgeräten (Smartphones, Tablets) mittels zugehöriger Applikation genutzt werden und bietet zahlreichen Exportformate (Excel, SPSS, SAS, R, JSON etc.).

Die Pseudonymisierungsliste liegt passwortgeschützt auf einer geschützten elektronischen Datenbank, nur unverblindete Mitarbeiterinnen und Mitarbeiter des Studienteams haben Zugriff. Zur Überprüfung der korrekten Übertragung der Behandlungsdaten aus der Krankenakte in die verschlüsselte Studiendatenbank dürfen bevollmächtigte Personen Einblick in die persönlichen Krankheitsdaten nehmen, die mit der Studie im Zusammenhang stehen. Alle beteiligten Mitarbeiter unterliegen der Schweigepflicht.

Rechtsgrundlage für die Verarbeitung sind Art. 6 Abs. 1 lit. a und Art. 9 Abs. 2 lit. a Datenschutz-Grundverordnung (DSGVO) in Verbindung mit der Einwilligung der/des Proband*in.

Für die Erhebung, Speicherung, Nutzung und Weitergabe der Daten ist die ausdrückliche Zustimmung durch Unterzeichnung der Einwilligungserklärung zum Datenschutz erforderlich.

Die Forschungsergebnisse aus der Studie werden in anonymisierter Form in Fachzeitschriften oder in wissenschaftlichen Datenbanken veröffentlicht. Die Verarbeitung der pseudonymisierten Daten erfolgt auf Erhebungsbögen und elektronischen Datenträgern.

Die Daten werden 10 Jahre nach Beendigung oder Abbruch der Studie aufbewahrt. Sie sind in elektronischer Form gegen unbefugten Zugriff geschützt und werden gelöscht, sobald sie für den Zweck der Datenverarbeitung im Rahmen der Studie nicht mehr benötigt werden, spätestens nach 10 Jahren.

Die im Verlauf dieser Studie gewonnenen Informationen werden ausschließlich in der EU, dem Europäischen Wirtschaftsraum oder Ländern mit vergleichbarem Datenschutzniveau verarbeitet.

Die Teilnehmenden können Ihre Einwilligung jederzeit ohne Angabe von Gründen schriftlich oder mündlich widerrufen, ohne dass ihnen daraus ein Nachteil entsteht. Wenn sie ihre Einwilligung widerrufen, werden keine weiteren Daten mehr erhoben. Die bis zum Widerruf erfolgte Datenverarbeitung bleibt jedoch rechtmäßig.

Sie können auch jederzeit Auskunft über Ihre gespeicherten Daten anfordern sowie die Überlassung einer kostenlosen Kopie verlangen und haben das Recht, fehlerhafte Daten berichtigen zu lassen. Sie können auch jederzeit verlangen, dass Ihre Daten gelöscht oder anonymisiert werden, so dass ein Bezug zu Ihrer Person nicht mehr hergestellt werden kann.

Diese Rechte sind nach § 13 des Landesdatenschutzgesetzes (LDSG) bzw. § 27 des Bundesdatenschutzgesetzes (BDSG) insoweit beschränkt, als diese Rechte voraussichtlich die Verwirklichung der jeweiligen Forschungszwecke unmöglich machen oder ernsthaft beeinträchtigen und die Beschränkung für die Erfüllung der jeweiligen Forschungszwecke notwendig ist.

Da die Studie in gemeinsamer Verantwortung des RBK und des UKT durchgeführt wird, liegt die Verantwortung für die Datenverarbeitung gemäß Artikel 26 der DSGVO sowohl bei der Robert Bosch Krankenhaus GmbH, Auerbachstr. 110, 70376 Stuttgart, Tel.: +49 711 8101-0 als auch beim Universitätsklinikum Tübingen, rechtsfähige Anstalt des öffentlichen Rechts der Universität Tübingen, Geissweg 3, 72076 Tübingen, Tel.: 07071 29-0, service@med.uni-tuebingen.de. Verantwortliche(r) für die Datenverarbeitung in dieser Studie sind die Dr. Marcela Winkler und Dr. Mirela Bilc. Bei Fragen zur Nutzung oder Verarbeitung Ihrer Daten wenden Sie sich bitten an diese.

Sollten Bedenken oder Beschwerden im Hinblick auf den Datenschutz bestehen oder die Rechte gemäß Art. 15ff. DSGVO wahrgenommen werden wollen, kann sich an folgenden Kontakte gewendet werden: Datenschutzbeauftragter der RBK, Robert Bosch Krankenhaus, Auerbachstr. 110, 70376 Stuttgart, E-Mail: datenschutzbeauftragter@rbk.de oder

Universitätsklinikum Tübingen, Datenschutzbeauftragter, Geissweg 3, 72076 Tübingen, Tel.: 07071 29-87667, E-Mail: Datenschutz@med.uni-tuebingen.de. Außerdem besteht das Recht sich bei der zuständigen Aufsichtsbehörde für den Datenschutz zu beschweren (Landesbeauftragter für den Datenschutz und die Informationsfreiheit in Baden-Württemberg, Postfach 10 29 32, 70025 Stuttgart, Tel.: 0711 / 61 55 41 - 716, Mail: Poststelle@lfdi.bwl.de).

12. Telemedizinische Verfahren

In Rahmen der vorliegenden Studie kommen keine telemedizinischen Verfahren zum Einsatz. Studienrelevante Erhebungen und Interventionen erfolgen im persönlichen Kontakt zwischen den Studienteilnehmenden und dem verantwortlichen Studienpersonal vor Ort. Eine Fernübertragung von medizinischen Daten ist nicht vorgesehen. Die Erhebung der studienbezogenen Fragebogendaten erfolgt in Papierform.

13. Bioproben

In Rahmen der vorliegenden Studie werden keine Bioproben entnommen oder gesammelt. Alle erforderlichen Daten werden ausschließlich aus den Patientenakte-Daten und der Auswertung der Fragebögen erhoben.

14. Unterschrift Studienleiter/in

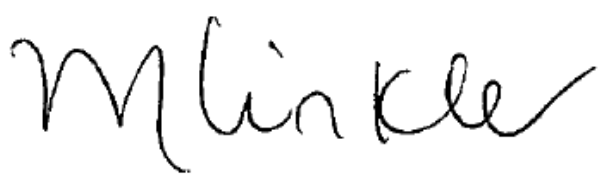

15. Literaturverzeichnis

1. Krebs in Deutschland für 2019/2020. Berlin: Zentrum für Krebsregisterdaten, GEK/D, Robert Koch Institut; 2023.
2. Tscheulin DK, Helmig B. Patientenzufriedenheitsmessungen im Krankenhaus. In: Albach H, Backes-Gellner U, editors. Krankenhausmanagement. Wiesbaden: Gabler Verlag; 2000. p. 105 - 22.
3. Aust H, Eberhart L, Sturm T, Schuster M, Nestoriuc Y, Brehm F, et al. A cross-sectional study on preoperative anxiety in adults. J Psychosom Res. 2018;111:133-9.
4. Hernández-Palazón J, Fuentes-García D, Falcón-Araña L, Roca-Calvo MJ, Burguillos-López S, Doménech-Asensi P, et al. Assessment of Preoperative Anxiety in Cardiac Surgery Patients Lacking a History of Anxiety: Contributing Factors and Postoperative Morbidity. J Cardiothorac Vasc Anesth. 2018;32(1):236-44.
5. Stamenkovic DM, Rancic NK, Latas MB, Neskovic V, Rondovic GM, Wu JD, et al. Preoperative anxiety and implications on postoperative recovery: what can we do to change our history. Minerva Anestesiol. 2018;84(11):1307-17.
6. Lorenz S. Die Prämedikationsambulanz: Analyse der Prozessqualität, Patientenzufriedenheit und Präoperativen Angst. Wiesbaden: Springer VS; 2023. 75-6 p.
7. Williams JB, Alexander KP, Morin JF, Langlois Y, Noiseux N, Perrault LP, et al. Preoperative anxiety as a predictor of mortality and major morbidity in patients aged >70 years undergoing cardiac surgery. Am J Cardiol. 2013;111(1):137-42.
8. Kang D, Lee JK, Kim N, Kim S, Lee SK, Lee JE, et al. Effect of mind and body education on quality of life among young breast cancer patients: a randomized controlled trial. Support Care Cancer. 2022;30(1):721-9.
9. Mishra A, Nair J, Sharan AM. Coping in Post-Mastectomy Breast Cancer Survivors and Need for Intervention: Systematic Review. Breast Cancer (Auckl). 2023;17:11782234231209126.
10. Jänig W, Birbaumer N. Physiologische Grundlagen von Emotion und Motivation. In: Brandes R, Lang F, Schmidt RF, editors. Physiologie des Menschen. 32. Auflage. Berlin: Springer; 2019. p. 848-63.
11. Dangor AA, Lam AM. Anesthesia for cerebral aneurysm surgery. Neurosurg Clin N Am. 1998;9(4):647-59.
12. Priebe HJ. Pharmacological modification of the perioperative stress response in noncardiac surgery. Best Pract Res Clin Anaesthesiol. 2016;30(2):171-89.
13. Adams HA, Hempelmann G. [The endocrine stress reaction in anesthesia and surgery--origin and significance]. Anasthesiol Intensivmed Notfallmed Schmerzther. 1991;26(6):294-305.
14. Iwasaki M, Edmondson M, Sakamoto A, Ma D. Anesthesia, surgical stress, and "long-term" outcomes. Acta Anaesthesiol Taiwan. 2015;53(3):99-104.
15. Powell R, Scott NW, Manyande A, Bruce J, Vögele C, Byrne-Davis LM, et al. Psychological preparation and postoperative outcomes for adults undergoing surgery under general anaesthesia. Cochrane Database Syst Rev. 2016;2016(5):Cd008646.
16. Shebl M, Toraih E, Shebl M, Tolba A, Ahmed P, Banga H, et al. Preoperative anxiety and its impact on surgical outcomes: A systematic review and meta-analysis. Journal of Clinical and Translational Science. 2025;9.
17. Wilson CJ, Mitchelson AJ, Tzeng TH, El-Othmani MM, Saleh J, Vasdev S, et al. Caring for the surgically anxious patient: a review of the interventions and a guide to optimizing surgical outcomes. The American Journal of Surgery. 2016;212(1):151-9.
18. Kahveci S, Taylan S. The Effect of Nonpharmacological Methods on Preoperative Anxiety in Breast Surgery Patients: Meta-analysis. J Perianesth Nurs. 2024.
19. Tong QY, Liu R, Zhang K, Gao Y, Cui GW, Shen WD. Can acupuncture therapy reduce preoperative anxiety? A systematic review and meta-analysis. J Integr Med. 2021;19(1):20-8.
20. Olshan-Perlmutter M, Carter K, Marx J. Auricular acupressure reduces anxiety and burnout in behavioral healthcare. Appl Nurs Res. 2019;49:57-63.
21. Spielberger C. State-trait anxiety inventory for adults. Mind Garden. 1983.

22. Berth H, Petrowski K, Balck F. The Amsterdam Preoperative Anxiety and Information Scale (APAIS) - the first trial of a German version. Psychosoc Med. 2007;4:Doc01.
23. Keller F., Kühner, C., Alexandrowicz, R. W., Voderholzer, U., Meule, A., Fegert, J. M., … Hautzinger, M. (2022). Zur Messqualität des Beck-Depressionsinventars (BDI-II) in unterschiedlichen klinischen Stichproben: Eine Item-Response-Theorie Analyse. Zeitschrift Für Klinische Psychologie Und Psychotherapie, *51(3–4)*, 234–246.
24. Madsen BK, Zetner D, Møller AM, Rosenberg J. Melatonin for preoperative and postoperative anxiety in adults. Cochrane Database Syst Rev. 2020;12(12):Cd009861.
25. Abdul-Latif MS, Putland AJ, McCluskey A, Meadows DP, Remington SAM. Oral midazolam premedication for day case breast surgery, a randomised prospective double-blind placebo-controlled study. Anaesthesia. 2001;56(10):990-4.
26. Harris, P. A., Taylor, R., Thielke, R., Payne, J., Gonzalez, N., & Conde, J. G. (2009). Research electronic data capture (REDCap)—a metadata-driven methodology and workflow process for providing translational research informatics support. Journal of biomedical informatics, 42(2), 377-381.

English (translation)

Study Protocol for Career Counseling

for submission to the Ethics Committee of the Baden-Württemberg State Medical Association

Option A: Prospective Study

Stuttgart,

Date: December 15, 2025

Table of Contents

Structured synopsis of the study 4
1. Project title, version number, version date 6
2. Responsibilities 6
3. Scientific Background 6
4. Project Objectives 9
5. Endpoints/Outcome Measures 9
6. Study Population 10
7. Methodology and Implementation 11
7.1 Study Design 11
7.2 Intervention 11
7.3 Data Collected 12
7.4 Recording and Assessment of Adverse Events 13
7.5 Documentation of Adverse Events 13
7.6 Study Protocol 13
8. Radiation Therapy 14
9. Benefit-Risk Assessment 14
10. Biometrics 15
11. Data Management and Data Protection 16
12. Telemedicine Procedures 18
13. Biological Samples 18
14. Signature of the Principal Investigator 18
15. Bibliography 19

Structured Summary of the Study

Version & Date	Version 3, December 15, 2025
Applicant	Marcela Winkler, M.D.<br>Auerbachstraße 110, 70376 Stuttgart<br>+49 711 8101-2073<br>marcela.winkler@rbk.de
Principal Investigator	Marcela Winkler, M.D.<br>Auerbachstraße 110, 70376 Stuttgart<br>+49 711 8101-2073<br>marcela.winkler@rbk.de
Study Title	Ear Acupressure to Reduce Perioperative Anxiety and Pain During Therapeutic Breast Surgery Following an Initial Diagnosis of Breast Cancer
Acronym	**ROSA -** Reduction of Surgery-Related Pain and Anxiety in Breast Cancer
Topic/Clinical Picture	A cancer diagnosis, upcoming treatments, and potential physical changes resulting from surgery place a significant burden on patients. Approximately 60–80% of patients report preoperative anxiety prior to breast surgery, which can negatively impact not only their well-being but also the course of treatment.<br>The NADA protocol, a standardized form of ear acupuncture, was originally developed for the treatment of addiction. Its benefits for cancer patients have not yet been extensively studied; however, experience from other fields suggests promising potential in oncology.
Objective	The aim of this study is to investigate the effect of ear acupressure on perioperative anxiety and pain in patients with a first-time breast cancer diagnosis who are undergoing therapeutic breast surgery.<br>Our hypothesis is that ear acupressure according to the NADA protocol reduces preoperative anxiety and postoperative pain.
Study Design	After confirming eligibility criteria, participants will be randomized into two groups: an intervention group and a control group. The target parameters will be assessed at the following time points: baseline (T0), immediately before being taken to the operating room (T1), postoperatively on the ward (T2), and daily until discharge (T3–Tx). Three months after surgery, a questionnaire regarding long-term treatment preferences will be mailed to participants (T-Follow-up).
Study-Related Interventions	The **intervention group** receives ear acupressure according to the NADA protocol. Before surgery, patients receive acupuncture patches applied to five specified points on the ear. These patches remain on the skin for five days. Patients are asked to press or massage the beads in the patch three times a day.<br>The **control group** receives standardized preoperative care. To increase motivation to participate, patients in the control group are offered NADA acupressure after discharge.
Study Design	Prospective randomized controlled trial<br>Due to the nature of the ear acupressure intervention, patients and study staff cannot be blinded. Statisticians are blinded.
Patient Population	Inclusion criteria:<br>• Age > 18 years<br>• Female

	• Breast cancer Patients who undergo therapeutic breast surgery at Robert Bosch Hospital following their initial diagnosis<br>• Written informed consent<br>Exclusion criteria:<br>• An anxiety disorder newly diagnosed within the last 12 months<br>• A mental state that, in the opinion of the investigator, precludes the patient from participating in the study<br>• Use of hearing aids<br>• Skin irritation or eczema in the ear<br>• Pregnancy<br>• Insufficient knowledge of German<br>• Unwillingness to allow the storage and sharing of personal medical data as part of the protocol<br>• Missing or incomplete informed consent form<br>• Receipt of acupuncture treatment (including NADA ear acupuncture or acupressure) within the last three months
Endpoints	Primary endpoint:<br>• Anxiety level immediately before surgery (STAI-s)<br>Secondary endpoints:<br>• Pain (NRS, medication)<br>• Postoperative nausea and vomiting (patient records)<br>• Postoperative complications (patient records)<br>• Preoperative anxiety (APAIS)<br>• State Anxiety (STAI-t)<br>• Adverse events<br>• Treatment Selection
Sample Size Calculation Sample Size	An MCID of 8 on the STAI corresponds to d = 0.8 (f = 0.4). For a covariance analysis with the intervention group as the between-subjects factor and baseline as the covariate, a two-sided significance level of 5%, and a power of 80%, 52 patients are required. Taking into account a dropout rate of 20%, n = 66 participants will be enrolled in the study and randomized 1:1.
Study Duration	The study duration per patient includes the entire inpatient stay (3 days on average) as well as a subsequent three-month follow-up period.<br>The total study duration is 18 months.
Number of Participating Centers	1 study center<br>Robert Bosch Hospital<br>Auerbachstr. 110<br>70376 Stuttgart

16. Project title, version number, version date

Project Title:	Ear Acupressure to Reduce Perioperative Anxiety and Pain During Therapeutic Breast Surgery Following an Initial Diagnosis of Breast Cancer
Acronym	**ROSA** – Reduction of Surgery-Related Pain and Anxiety in Breast Cancer
Version number:	3
Version Date:	December 15, 2025

17. Responsibilities

Applicant and Contact Person for the Ethics Committee	Dr. Marcela Winkler
Principal Investigator	Dr. Marcela Winkler<br>Auerbachstraße 110, 70376 Stuttgart<br>+49 711 8101-2073<br>marcela.winkler@rbk.de
Co-Investigator	Dr. Mirela-Ioana Bilc<br>Auerbachstraße 112, 70376 Stuttgart<br>Phone +49 711 8101-3766<br>mirela.bilc@bosch-health-campus.com
Study Physician	Dr. Marcela Winkler
Other participating clinical/scientific personnel	Prof. Dr. Holger Cramer<br>Prof. Dr. Georg Sauer

18. Scientific Background

With an incidence of approximately 70,000 new cases per year, breast cancer is the most common cancer among women in Germany (1). Only 1% of new diagnoses involve men. Surgical intervention is one of the three main pillars of treatment, alongside drug therapy and radiation therapy.

In addition to uncertainty about the course of the disease and the necessity of surgical treatment, the period immediately preceding surgery is a situation particularly often associated with anxiety (2). Preoperative anxiety is reported by 60–80% of patients, although the size, duration, and invasiveness of the surgery are not decisive factors in determining the extent of preoperative anxiety (3–5). The anxiety experienced in this situation is significantly more influenced by the subjective perception of threat than by the objective extent of the danger (6, 7). In women, breast cancer surgery often leads to changes in body image, which causes additional psychological stress and anxiety (8, 9).

As a basic emotion, anxiety has direct somatic effects on our autonomic and neuroendocrine systems through activation of the hypothalamic-pituitary-adrenal axis (HPA) and an increase in sympathetic tone (10). Anxiety is further influenced by sensory input from the sensory organs, which reaches the amygdala via the thalamus and thalamic-amygdalar pathways. The amygdala plays a crucial role in processing this sensory input and, through potential transmission to the anterior pituitary, also in activating the HHNR axis. The activation of the sympathetic nervous system—which is evolutionarily necessary in flight-or-fight situations and is triggered by anxiety—leads, via the catecholamines norepinephrine and epinephrine, to an increase in blood pressure, heart rate, and cardiac output, among other effects (10).

This increase leads, particularly in patients with pre-existing cardiac conditions, to an elevated risk of cardiac ischemia, a perioperative heart attack, or stroke; and in patients with pre-existing aneurysms, it leads to increased mortality due to the risk of rupture (11, 12).

In the context of tumor resection, the anxiety response is also relevant due to a reduction in immune function associated with increased cortisol release. This cortisol-induced immunosuppression thus leads not only to an increased risk of postoperative infections and neoplasms but also to an increased risk of metastasis due to reduced elimination of preoperative micrometastases (13, 14).

Another parameter proven to be influenced by preoperative anxiety is the postoperative pain experienced by patients, which, along with perceived anxiety, contributes to additional suffering in patients undergoing surgery (15). A recent meta-analysis has demonstrated significant associations between preoperative anxiety and an increased need for anesthetics and analgesics (16).

Reducing patients' anxiety can thus lead to an overall better postoperative outcome through improved hemodynamic stability, enhanced immune function and wound healing, and reduced postoperative pain (6).

There are many different therapeutic options, both pharmacological and non-pharmacological; however, when choosing the best approach, it is crucial to consider the patient's anxiety level and the overall clinical situation (17). Acupuncture appears to be a promising, low-risk option for reducing preoperative anxiety. A recent meta-analysis of non-pharmacological therapies, including aromatherapy, music, and acupuncture, found a moderate effect size in reducing preoperative anxiety in female patients undergoing breast surgery (18). A meta-analysis (19) of 12 randomized controlled trials involving a total of 916 patients showed that acupuncture can significantly reduce preoperative anxiety, as measured by the State-Trait Anxiety Inventory Scale (STAI-S) and the visual analog scale (VAS). However, the quality of the evidence was low to moderate, suggesting that further high-quality studies are needed to confirm these findings. Additionally, different acupuncture or acupressure points were used in these studies, which makes comparisons difficult.

One method that could be promising in this regard is the NADA protocol, a standardized form of ear acupuncture originally developed as an adjunct therapy in addiction medicine. However, it has demonstrated its effectiveness in various contexts, including disaster medicine (e.g., following the 9/11 terrorist attacks or Hurricane Katrina) and in the care of traumatized individuals. Particularly relevant to perioperative medicine is the stress-regulating effect of the NADA protocol. It is hypothesized that by modulating the autonomic nervous system—specifically by reducing sympathetic overactivity and strengthening the parasympathetic system—anxiety and stress-related physiological reactions can be alleviated (20).

However, the NADA protocol has so far been insufficiently studied specifically in the context of oncology, although experience from other therapeutic indications suggests a potential benefit for oncology patients. Initial studies and case reports show positive effects in oncology patients, particularly in those with breast and prostate cancer who suffer from side effects of endocrine therapies, sleep disturbances, fatigue, anxiety, and depressive symptoms.

However, there have been very few systematic studies to date on the application of the NADA protocol in an oncological context, particularly in the perioperative setting. Previous studies

point to possible positive effects; however, well-designed randomized controlled trials are needed to evaluate the exact efficacy and optimal scope of application in this setting.

19. Project Objectives

This study investigates the effect of ear acupressure on perioperative anxiety and pain in patients following a first diagnosis of breast cancer who are undergoing therapeutic breast surgery. Specifically, we will examine the effect of the NADA acupressure protocol on preoperative anxiety levels and postoperative pain. We will also conduct an exploratory analysis to determine whether this intervention influences treatment selection in the 3 months following surgery.

20. Endpoints/Outcome Measures

The primary outcome measure of this study is the level of anxiety immediately before surgery, measured using the State-Trait Anxiety Inventory (STAI-s). Secondary outcome measures include pain intensity, postoperative nausea and vomiting (PONV), postoperative complications, specific preoperative anxiety, and adverse events. Treatment choice will be assessed after a 3-month follow-up using a questionnaire mailed to participants.

Our hypothesis is that ear acupressure according to the NADA protocol reduces preoperative anxiety and postoperative pain.

21. Study Population

For this study, we will recruit N = 66 breast cancer patients who will undergo therapeutic breast surgery at Robert Bosch Hospital following their initial diagnosis. Recruitment will take place directly through the Department of Naturopathy and Integrative Medicine at Robert Bosch Hospital.

As part of routine care, the *study physician* will inform potential participants about the opportunity to participate in the study. If interested, patients will receive the patient information sheet for the study. The *study physician* provides patients with a comprehensive explanation of the study and answers any questions that arise in detail and in an understandable manner. Only after this explanation is the written informed consent form for study participation obtained. Participation is voluntary and may be withdrawn at any time without providing a reason, without any disadvantage to the patients.

Following consent, the study physician conducts a medical screening to determine whether the patient meets the eligibility criteria for study participation. The decision regarding eligibility is based on the following criteria:

Inclusion Criteria:

- Age > 18 years
- Female
- Breast cancer Patients who undergo therapeutic breast surgery at Robert Bosch Hospital following their initial diagnosis
- Written informed consent

Exclusion criteria:

- Anxiety disorder newly diagnosed within the last 12 months
- A mental state that, in the opinion of the investigator, precludes the patient from participating in the study
- Use of hearing aids
- Skin irritation or eczema of the ear
- Pregnancy
- Insufficient knowledge of German
- Unwillingness to allow the storage and sharing of personal medical data as part of the protocol
- Missing or incomplete informed consent form
- Receipt of acupuncture treatment (including NADA ear acupuncture or acupressure) within the last three months

22. Methodology and Implementation

7.2 Study Design

This study is a prospective randomized controlled trial comparing the effect of ear acupressure according to the NADA protocol with a control group receiving standard preoperative care.

Due to the nature of the ear acupressure intervention, patients and study staff cannot be blinded. Statisticians will be blinded.

7.2 Intervention

Intervention Group: NADA Ear Acupressure + Standard Care

Study participants in the intervention arm receive standardized ear acupressure treatment according to the NADA protocol in addition to standard care. This is performed using special acupuncture patches that apply pressure to specific points on the ear via a small bead. As part of the NADA protocol, five specified points on both ears are treated according to a standardized approach: Shen Men, Kidney, Lung, Liver, and Autonomic Nervous System(24) . To prevent contact allergies, gold-coated beads will be used.

Patients receive the NADA protocol prior to surgery. They are asked to leave the patches on their ears for 5 days after application and to press or massage the beads themselves three times daily according to the provided instructions. At the start of the intervention, patients receive an information sheet with application instructions and behavioral guidelines (see Appendix 1).

NADA ear acupressure is a non-invasive and low-risk intervention. Furthermore, the protocol is performed exclusively by NADA-certified physicians, medical assistants, or nursing staff

Control Group: Standard Care

Patients in the control group receive standard preoperative care. On the day of discharge from the hospital, they are offered NADA acupressure.

7.3 Data Collection

General sociodemographic data will be collected at the start of the study using a questionnaire. These questions include, among other things, marital status, ethnic background, employment status, and days lost to illness.

<u>Primary Outcome Measure:</u>

The primary outcome measure of this study is the level of anxiety immediately before surgery, as measured by the State-Trait Anxiety Inventory (STAI-s)(21) .

<u>Secondary Outcome Measures</u>

Pain: Pain intensity is assessed using a Numerical Rating Scale, and the patient's medication is recorded from the medical record.

Postoperative nausea and vomiting (PONV) as well as the use of antiemetics are extracted from the patient's medical record. In addition, postoperative complications (e.g., infections, postoperative bleeding) are recorded.

Preoperative anxiety is specifically assessed using the Amsterdam Preoperative Anxiety and Information Scale (APAIS)(22) .

Trait anxiety is assessed using the State-Trait Anxiety Inventory (trait measure)(21) .

The severity of depression is assessed preoperatively using the Beck Depression Inventory (BDI-II) (23).

Adverse events: To assess the safety of the interventions, adverse events are systematically recorded throughout the entire intervention by specifically asking about them and documenting them.

Treatment selection is assessed after a 3-month follow-up using a questionnaire mailed to participants.

To assess the safety of the interventions, adverse events are systematically recorded after the intervention by specifically asking about them and documenting them.

7.4 Recording and Assessment of Adverse Events

Adverse events are illnesses, signs of illness, or symptoms that occur or worsen after the patient's enrollment in the study, either during or shortly after the intervention. The severity is assessed as follows: mild, moderate, severe, life-threatening.

A causality assessment must be performed for each event: no association, possible association, probable association, definite association.

According to the GCP Regulation (GCP-V §3), "serious" is defined as follows: A serious adverse event or serious adverse reaction is any event that is: fatal or life-threatening; requires hospitalization or prolongation of hospitalization; results in permanent or severe disability or invalidity; or results in a congenital anomaly or birth defect

7.5 Documentation of Adverse Events

Every adverse event must be documented if, in the investigator's opinion, there is a causal relationship with the intervention. The documentation must include the type of event, severity, onset, duration, causality, and implications for the study.

Related signs and symptoms should be grouped into a single condition. The "Adverse Events—Study Patient" form is available for documentation. All adverse events must be monitored until they subside or stabilize.

7.6 Study Protocol

Data are collected at the following time points: baseline (T0), immediately before admission to the operating room (T1), on the ward after surgery (T2), and daily until discharge (T3–Tx). After 3 months, a questionnaire regarding treatment selection is mailed to participants ($T_{Follow-up}$). The data collection schedule is shown in Table 1.

Table 1: Data Collection Schedule

	T0	T1	T2	T3–Tx	$T_{Follow-up}$
STAI-s	x	x	x	x	
NRS Pain	x	x	x	x	
PONV			x	x	
Postoperative Complications			x	x	
APAIS	x				
STAI-t	x				
BDI	x				
AE		x	x	x	
Therapy Selection Questionnaire					x

The study is scheduled to run for 18 months. Taking into account the number of patients who undergo breast surgery at the RBK each year (approximately 200) and a recruitment rate of about 30%, we expect to reach our target sample size of N = 66 in approximately 12 months.

23. Radiation Exposure

No radiation exposure is planned as part of this clinical trial. Neither diagnostic nor therapeutic procedures involving radiation exposure for the participants will be used.

24. Benefit-Risk Assessment

Participation in this study may offer potential individual benefits to some participants, particularly through a possible reduction in preoperative anxiety and pain. In addition, a group or external benefit is expected in the form of scientific knowledge gained , which may contribute to improving future preoperative treatment strategies.

The burdens and risks associated with study participation are considered to be low. Ear acupressure is a non-invasive intervention with an extremely low risk profile. Skin irritation may occasionally occur; however, no other adverse side effects have been reported. Surveys using questionnaires or rating scales are also completely non-invasive and pose no study-specific risks; each survey takes only a few minutes to complete.

From a medical-ethical perspective, conducting the study is justifiable based on the current state of knowledge, as the potential benefits outweigh the expected minimal risks. An individual participant's involvement may be terminated in the event of unexpected side effects, withdrawal of consent, or for medical reasons. The entire study may be terminated prematurely in the event of unforeseen safety concerns, serious complications, or insufficient recruitment.

25. Biometrics

Group differences of at least 8 points on the STAI can be considered minimally clinically significant(24) . Assuming a standard deviation of SD = 10 corresponding to this population(25) , this results in an effect size of d = 0.8. Based on a corresponding effect size of f = 0.4, a covariance analysis with the intervention group as the between-subjects factor and baseline as the covariate, a two-sided significance level of 5%, and a power of 80% yields a required sample size of 52 patients in total. Taking into account a dropout rate of 20%, n = 66 female participants will be enrolled in the study and assigned to the study groups in a 1:1 ratio. Group assignment will be performed using block randomization with varying block lengths and the type of breast surgery as the stratification variable. Randomization is performed using a

computer-generated randomization list via the REDCap (Research Electronic Data Capture) data collection tool (26). Randomization takes place after the baseline assessment at T0. The primary outcome measure is analyzed according to the intention-to-treat principle. Missing values are imputed using multiple imputation. The primary outcome measure is analyzed confirmatorily using a univariate analysis of covariance (ANCOVA), with the intervention group (NADA ear acupressure vs. control) as the between-subjects factor and the baseline measurement as a covariate. The secondary endpoints are evaluated exploratorily using the same models. The effects of trait anxiety and situational preoperative anxiety are examined using moderation analysis.

As a measure of the safety and acceptability of the intervention, the number of patients who experience serious adverse events or non-serious adverse events, or who are lost to follow-up during the study period, will be recorded and compared between groups using absolute and relative frequencies.

26. Data Management and Data Protection

Personal data is collected and processed as part of this study. The data is documented and archived in pseudonymized form in a secure electronic database (REDCap), to which only authorized staff members—including doctoral students bound by professional and data confidentiality obligations—have access.

The data collection tool *REDCap* (Research Electronic Data Capture) is used as the data platform. *REDCap* is a free, secure, and browser-based software for creating and managing databases; it was developed at Vanderbilt University and is managed and distributed by the *REDCap Consortium* (Harris et al., 2009). *REDCap* can be used on mobile devices (smartphones, tablets) via a dedicated app and offers numerous export formats (Excel, SPSS, SAS, R, JSON, etc.).

The pseudonymization list is stored in a password-protected, secure electronic database; only unblinded members of the study team have access to it. To verify the correct transfer of treatment data from the medical record to the encrypted study database, authorized persons may review the personal medical data related to the study. All participating staff members are bound by confidentiality obligations.

The legal basis for the processing is Article 6(1)(a) and Article 9(2)(a) of the General Data Protection Regulation (GDPR) in conjunction with the participant’s consent.

The collection, storage, use, and disclosure of the data require explicit consent by signing the data protection consent form.
The research results from the study will be published in anonymized form in academic journals or scientific databases. The processing of pseudonymized data takes place on survey forms and electronic data carriers.
The data will be retained for 10 years after the study's completion or termination. It is protected against unauthorized access in electronic form and will be deleted as soon as it is no longer needed for the purpose of data processing within the scope of the study, at the latest after 10 years.
The information collected during this study will be processed exclusively within the EU, the European Economic Area, or countries with comparable data protection standards.
Participants may withdraw their consent at any time, in writing or verbally, without providing a reason and without suffering any disadvantage as a result. If they withdraw their consent, no further data will be collected. However, data processing that took place prior to the withdrawal remains lawful.
You may also request information about your stored data at any time, as well as request a free copy, and you have the right to have incorrect data corrected. You may also request at any time that your data be deleted or anonymized so that it can no longer be linked to you personally.
These rights are restricted under Section 13 of the State Data Protection Act (LDSG) and Section 27 of the Federal Data Protection Act (BDSG) to the extent that these rights are likely to make the achievement of the respective research purposes impossible or seriously impair it, and the restriction is necessary for the fulfillment of the respective research purposes.
Since the study is being conducted under the joint responsibility of the RBK and the UKT, responsibility for data processing pursuant to Article 26 of the GDPR lies with both Robert Bosch Krankenhaus GmbH, Auerbachstr. 110, 70376 Stuttgart, Tel.: +49 711 8101-0, and the University Hospital of Tübingen, a public-law institution with legal capacity affiliated with the University of Tübingen, Geissweg 3, 72076 Tübingen, Tel.: 07071 29-0, service@med.uni-tuebingen.de. Dr. Marcela Winkler and Dr. Mirela Bilc are the data controllers for this study. If you have any questions regarding the use or processing of your data, please contact them.
If you have any concerns or complaints regarding data protection, or if you wish to exercise your rights under Art. 15 et seq. of the GDPR, you may contact the following: Data Protection Officer of the RBK, Robert Bosch Hospital, Auerbachstr. 110, 70376 Stuttgart,

email:datenschutzbeauftragter@rbk.de or Tübingen University Hospital, Data Protection Officer, Geissweg 3, 72076 Tübingen, Tel.: 07071 29-87667, Email:Datenschutz@med.uni-tuebingen.de . You also have the right to file a complaint with the competent data protection supervisory authority (State Commissioner for Data Protection and Freedom of Information in Baden- -Württemberg, P.O. Box 10 29 32, 70025 Stuttgart, Tel.: 0711 / 61 55 41 - 716, Email:Poststelle@lfdi.bwl.de).

27. Telemedicine Procedures

No telemedicine procedures are used in this study. Data collection and interventions relevant to the study take place through in-person contact between the study participants and the responsible study staff on site. Remote transmission of medical data is not planned. Study-related questionnaire data is collected in paper form.

28. Biological samples

No biological samples will be collected or obtained as part of this study. All necessary data will be gathered exclusively from patient medical records and the analysis of the questionnaires.

29. Signature of the Principal Investigator

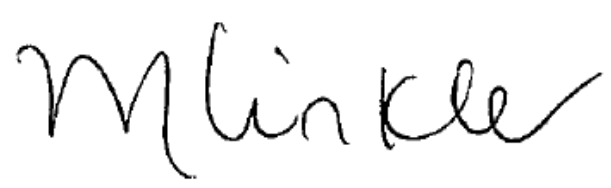

30. Bibliography

1. Cancer in Germany for 2019/2020. Berlin: Center for Cancer Registry Data, GEK/D, Robert Koch Institute; 2023.
2. Tscheulin DK, Helmig B. Measuring Patient Satisfaction in Hospitals. In: Albach H, Backes-Gellner U, editors. Hospital Management. Wiesbaden: Gabler Verlag; 2000. pp. 105–22.
3. Aust H, Eberhart L, Sturm T, Schuster M, Nestoriuc Y, Brehm F, et al. A cross-sectional study on preoperative anxiety in adults. J Psychosom Res. 2018;111:133–9.
4. Hernández-Palazón J, Fuentes-García D, Falcón-Araña L, Roca-Calvo MJ, Burguillos-López S, Doménech-Asensi P, et al. Assessment of Preoperative Anxiety in Cardiac Surgery Patients Lacking a History of Anxiety: Contributing Factors and Postoperative Morbidity. J Cardiothorac Vasc Anesth. 2018;32(1):236-44.
5. Stamenkovic DM, Rancic NK, Latas MB, Neskovic V, Rondovic GM, Wu JD, et al. Preoperative Anxiety and Its Implications for Postoperative Recovery: What Can We Do to Change Our Approach? Minerva Anestesiol. 2018;84(11):1307-17.
6. Lorenz S. The Premedication Clinic: Analysis of Process Quality, Patient Satisfaction, and Preoperative Anxiety. Wiesbaden: Springer VS; 2023. 75-6 p.
7. Williams JB, Alexander KP, Morin JF, Langlois Y, Noiseux N, Perrault LP, et al. Preoperative anxiety as a predictor of mortality and major morbidity in patients aged >70 years undergoing cardiac surgery. Am J Cardiol. 2013;111(1):137-42.
8. Kang D, Lee JK, Kim N, Kim S, Lee SK, Lee JE, et al. Effect of mind-body education on quality of life among young breast cancer patients: a randomized controlled trial. Support Care Cancer. 2022;30(1):721–9.
9. Mishra A, Nair J, Sharan AM. Coping in Post-Mastectomy Breast Cancer Survivors and the Need for Intervention: A Systematic Review. Breast Cancer (Auckl). 2023;17:11782234231209126.
10. Jänig W, Birbaumer N. Physiological Foundations of Emotion and Motivation. In: Brandes R, Lang F, Schmidt RF, eds. Human Physiology. 32nd ed. Berlin: Springer; 2019. p. 848-63.
11. Dangor AA, Lam AM. Anesthesia for cerebral aneurysm surgery. Neurosurg Clin N Am. 1998;9(4):647-59.
12. Priebe HJ. Pharmacological Modification of the Perioperative Stress Response in Noncardiac Surgery. Best Pract Res Clin Anaesthesiol. 2016;30(2):171–89.
13. Adams HA, Hempelmann G. [The endocrine stress reaction in anesthesia and surgery—origin and significance]. Anasthesiol Intensivmed Notfallmed Schmerzther. 1991;26(6):294-305.
14. Iwasaki M, Edmondson M, Sakamoto A, Ma D. Anesthesia, surgical stress, and “long-term” outcomes. Acta Anaesthesiol Taiwan. 2015;53(3):99-104.
15. Powell R, Scott NW, Manyande A, Bruce J, Vögele C, Byrne-Davis LM, et al. Psychological preparation and postoperative outcomes for adults undergoing surgery under general anesthesia. Cochrane Database Syst Rev. 2016;2016(5):Cd008646.
16. Shebl M, Toraih E, Shebl M, Tolba A, Ahmed P, Banga H, et al. Preoperative anxiety and its impact on surgical outcomes: A systematic review and meta-analysis. Journal of Clinical and Translational Science. 2025;9.
17. Wilson CJ, Mitchelson AJ, Tzeng TH, El-Othmani MM, Saleh J, Vasdev S, et al. Caring for the surgically anxious patient: a review of interventions and a guide to optimizing surgical outcomes. The American Journal of Surgery. 2016;212(1):151-9.
18. Kahveci S, Taylan S. The Effect of Nonpharmacological Methods on Preoperative Anxiety in Breast Surgery Patients: Meta-analysis. J Perianesth Nurs. 2024.
19. Tong QY, Liu R, Zhang K, Gao Y, Cui GW, Shen WD. Can acupuncture therapy reduce preoperative anxiety? A systematic review and meta-analysis. J Integr Med. 2021;19(1):20-8.
20. Olshan-Perlmutter M, Carter K, Marx J. Auricular acupressure reduces anxiety and burnout in behavioral healthcare. Appl Nurs Res. 2019;49:57-63.
21. Spielberger C. State-Trait Anxiety Inventory for Adults. Mind Garden. 1983.

22. Berth H, Petrowski K, Balck F. The Amsterdam Preoperative Anxiety and Information Scale (APAIS)—the first trial of a German version. Psychosoc Med. 2007;4:Doc01.
23. Keller F., Kühner, C., Alexandrowicz, R. W., Voderholzer, U., Meule, A., Fegert, J. M., … Hautzinger, M. (2022). On the measurement quality of the Beck Depression Inventory (BDI-II) in different clinical samples: An item response theory analysis. Journal of Clinical Psychology and Psychotherapy, *51(3–4)*, 234–246.
24. Madsen BK, Zetner D, Møller AM, Rosenberg J. Melatonin for preoperative and postoperative anxiety in adults. Cochrane Database Syst Rev. 2020;12(12):Cd009861.
25. Abdul-Latif MS, Putland AJ, McCluskey A, Meadows DP, Remington SAM. Oral midazolam premedication for day-case breast surgery: a randomized, prospective, double-blind, placebo-controlled study. Anaesthesia. 2001;56(10):990-4.
26. Harris, P. A., Taylor, R., Thielke, R., Payne, J., Gonzalez, N., & Conde, J. G. (2009). Research Electronic Data Capture (REDCap)—a metadata-driven methodology and workflow process for providing translational research informatics support. Journal of Biomedical Informatics, 42(2), 377-381.
