# Supplementary material for: Auricular Acupressure for Perioperative Anxiety and Pain in Therapeutic Breast Surgery: Protocol for a Randomized Clinical Trial
Source: JMIR Res Protoc. 2026 Aug 21;15:e97469. doi: 10.2196/97469 (PMC13496320; doi:10.2196/97469)
Supplement: Supplementary file 2 [file resprot-v15-e97469-s002.docx]

**S2**

**PRECIS-2 tool evaluation of trial applicability**

PRECIS-2 scores for trial domains^1^

| Domain | Score | Rationale |
| --- | --- | --- |
| 1. Eligibility Criteria   *Who is selected to participate in the trial?* | 3 | Similarity between trials participants and those likely to be a candidate for intervention if it was provided in usual care is decreased by the use of specific exclusion criteria such as being diagnosed with an anxiety disorder within the previous 12 months; having in the opinion of the principal investigator, a psychological or mental condition precludes safe or appropriate participation in the study; unwillingness to permit storage and processing of personal health data as required by the study protocol; missing or incomplete informed consent; and prior acupuncture treatment (including NADA auricular acupuncture or acupressure) within the previous three months. |
| 2. Recruitment Path   *How are participants recruited into the trial?* | 5 | Participants are recruited from the clinic’s in-house patient pool when they attend routine appointments as part of usual care. No additional recruitment approaches are used. |
| 3. Setting   *Where is the trial being done?* | 4 | Trial is running in a very similar setting to which results are intended to be applied. PRECIS score was decreased as this is a trial restricted to single center, while the results should be applicable in other centers as well. |
| 4. Organisation intervention   *What expertise and resources are needed to deliver the intervention?* | 5 | Intervention is embedded within the context of usual organization of care with no additional healthcare staff and resources. |
| 5. Flexibility of experimental intervention – Delivery   *How should the intervention be delivered?* | 2 | Flexibility of intervention delivery is reduced by having a protocol-driven intervention with specific instructions on when, how and for how long to deliver the acupressure intervention. |
| 6. Flexibility of experimental intervention – Adherence   *What measures are in place to make sure participants adhere to the intervention?* | 2 | Flexibility of intervention adherence is reduced by having measures such as daily visits from study personnel to monitor compliance and side-effects. |
| 7. Follow up   *How closely are participants followed-up?* | 2 | PRECIS score was decreased as participants are followed-up intensively (daily measurements), which would not occur under usual care. |
| 8. Primary outcome   *How relevant is it to participants?* | 5 | Primary outcome is state anxiety prior to surgery, a patient-reported outcome measure (PROMs) of direct relevance to patients. Other PROMs include postoperative anxiety, pre- and postoperative pain, postoperative nausea and vomiting, specific surgery-related preoperative anxiety, trait anxiety and depressive symptoms. |
| 9. Primary analysis   *To what extent are all data included?* | 5 | Planned analysis is intention-to-treat using all available data. |

Note: 1 = Very explanatory, 2 = Rather explanatory, 3 = Equally pragmatic/explanatory, 4 = Rather pragmatic, 5 = Very pragmatic

^1^ Loudon K, Treweek S, Sullivan F, Donnan P, Thorpe K E, Zwarenstein M et al. The PRECIS-2 tool: designing trials that are fit for purpose *BMJ* 2015; 350 :h2147 doi:10.1136/bmj.h2147
